# Quality Analysis and Detection of Adulterants and Contaminations in Milk/Milk Powder by Raman Spectroscopy

**DOI:** 10.1111/1541-4337.70403

**Published:** 2026-01-24

**Authors:** B. Sudarshan Acharya, Sreerag Nair, Abdul Ajees Abdul Salam

**Affiliations:** ^1^ Manipal Institute of Applied Physics Manipal Academy of Higher Education Manipal, Udupi Karnataka India

**Keywords:** calibration transfer, chemometrics, dairy authentication, explainable artificial intelligence, food safety, hyperspectral Raman imaging, milk adulteration, portable sensors, Raman spectroscopy, surface‐enhanced Raman spectroscopy

## Abstract

Milk and milk powder are central to global nutrition, yet remain vulnerable to adulteration and contamination. Adulteration using water, urea, ammonium sulfate, thiocyanates, detergents, melamine, or compositional changes with whey and carbohydrate fillers undermines nutritional quality, reduces consumer confidence, and challenges regulatory control, particularly in infant formula products. A field‐ready analytical platform that is rapid, nondestructive, and capable of multi‐adulterant surveillance is urgently needed across diverse dairy matrices. This review consolidates advances in Raman spectroscopy for milk and milk powder authentication reported from 2015 to early 2025, covering conventional Raman, surface‐enhanced Raman spectroscopy (SERS), Fourier‐transform Raman, hyperspectral Raman imaging, confocal/mapping approaches, and portable systems. We critically evaluate preprocessing and chemometrics such as principal component analysis, partial least squares regression, and partial least squares discriminant analysis, as well as machine‐learning and deep‐learning pipelines for classification and quantification. Species‐specific applications including cow, buffalo, goat, camel, donkey, human breast milk (macronutrients, sex‐linked profiles, microplastics, antibiotics), and milk powder workflows are compared with attention to matrix effects, fluorescence interference, and validation practices. Raman enables chemically specific fingerprints of proteins, lipids, and carbohydrates, whereas common adulterants present diagnostic bands. SERS substrates routinely extend sensitivity to ppm–ppb levels and suppress fluorescence, supporting rapid detection of melamine, urea, ammonium sulfate, thiocyanates, benzoate, and selected antibiotics. Hyperspectral imaging provides spatially resolved maps, differentiating multi‐adulterant mixtures and thermo‐structural behavior in powders. Chemometric models achieve high accuracy for classification and concentration prediction, whereas deep‐learning architectures improve robustness under nonlinear matrix variation and instrument drift. Challenges persist in substrate reproducibility, calibration transfer, fluorescence in lipid‐rich systems, and detection of emerging adulterants and trace preservatives under field conditions. Future progress will hinge on multi‐excitation instruments with adaptive laser power control, universal SERS substrates integrating plasmonic metals, dielectric shells, and molecular recognition, and standard operating procedure grade preprocessing. Industrial reliability requires calibration‐transfer strategies, rigorous validation, and explainable artificial intelligence to link decisions to chemically meaningful features, supporting regulatory acceptance and auditability. Portable Raman and SERS systems can aid nutritional profiling and contaminant surveillance in breast milk, whereas Fourier‐transform Raman and hyperspectral imaging mitigate fluorescence and map heterogeneity in powders. Raman spectroscopy, augmented by SERS, hyperspectral imaging, and intelligent analytics, offers a rapid, nondestructive, label‐free, and scalable platform for dairy authentication. Continued innovation will enable real‐time, on‐site detection of single and multiple adulterants, strengthening consumer confidence, industrial quality assurance, and regulatory compliance while advancing global food safety.

**Abbreviations**
AgNP: silver nanoparticle
AuNP: gold nanoparticle
AI: artificial intelligence
AmS: ammonium sulfate
Au@AgNPs: gold–silver core–shell nanoparticles
Au@SiO2: silica‐coated gold nanoparticles
BA: benzoic acid
CNN: convolutional neural network
CNTs: carbon nanotubes
DCD: dicyandiamide
FT‐IR: fourier transform (FT)‐infrared spectroscopy
HSI: hyperspectral Raman imaging
HPLC: high‐performance liquid chromatography
LA: linoleic acid
LOD: limit of detection
LOQ: limit of quantification
ML: machine‐learning
MSC: multiplicative scatter correction
MS: mass spectrometry
NIR: near‐infrared spectroscopy
PCA: principal component analysis
PLS: partial least squares
PLS‐DA: partial least squares discriminant analysis
PLSR: partial least squares regression
*R* ^2^
: coefficient of determination
RMSEP: root mean square error of prediction
RPD: residual predictive deviation
SERS: surface‐enhanced Raman spectroscopy
SCN: thiocyanate
STC: sodium thiocyanate
SVM: support vector machine

## 1 Introduction

Raman spectroscopy has evolved into a versatile, rapid, and nondestructive vibrational spectroscopic technique capable of delivering chemically specific molecular “fingerprints” from complex food matrices (Chandra et al. 2024). By probing molecular vibrations through the inelastic scattering of monochromatic light, Raman spectroscopy enables direct compositional analysis with minimal sample preparation, even in heterogeneous systems where conventional optical techniques often face limitations (Boyaci et al. 2015; Li, Chang, et al. 2024; Li, Hussain, et al. 2024). Over the past decade, advances in laser sources, detector sensitivity, spectral preprocessing, and multivariate data analysis have significantly enhanced the robustness and analytical reliability of Raman‐based measurements. As a result, the technique has gained increasing attraction across food safety applications, including rapid pathogen screening, early spoilage detection via volatile degradation products such as ammonia and hydrogen sulfide, and identification of chemical residues in agricultural products (Li, Hussain, et al. 2024; Wang, Chen, et al. 2021).

Milk is among the most nutritionally complex and globally consumed food commodities. It provides high‐quality protein containing all essential amino acids, bioactive lipids, and a broad spectrum of vitamins and minerals essential for growth, metabolic regulation, immune function, and skeletal development (Gaffney‐Stomberg et al. 2009; Górska‐Warsewicz et al. 2019). Species‐dependent variations in protein composition, lipid profile, and mineral bioavailability influence digestibility, nutritional functionality, and processing behavior (Magan et al. 2021). Human milk is optimized for infant development, with a whey‐rich protein profile and elevated levels of long‐chain polyunsaturated fatty acids, whereas cow, goat, buffalo, and other ruminant milks are richer in casein and minerals, supporting industrial dairy production but presenting distinct nutritional and functional attributes (Andreas et al. 2015; Brockway et al. 2024). These intrinsic compositional differences underpin the growing need for accurate species identification, quality assurance, and authentication across the dairy value chain.

The global scale and economic significance of dairy production further amplify the importance of robust analytical monitoring. Annual milk production now approaches one billion tonnes across species, making dairy a cornerstone of food security, rural livelihoods, and international trade (Assan 2025). At the same time, milk's high value, perishability, and extended supply chains render it particularly vulnerable to economically motivated adulteration, including dilution, compositional mimicry, and the addition of chemical preservatives or nitrogen‐rich compounds to mask quality loss (Ionescu et al. 2023). Such practices compromise nutritional integrity, undermine consumer trust, and pose significant public health risks, reinforcing the need for reliable surveillance strategies. The public‐health and regulatory stakes associated with milk adulteration are substantial. The World Health Organization estimates that foodborne diseases cause approximately 600 million illnesses and 420,000 deaths annually, highlighting the need for rapid, matrix‐tolerant surveillance tools across dairy supply chains (Li, Hussain, et al. 2024). In response to major adulteration incidents, most notably melamine adulteration in infant formula, international regulators have defined stringent contaminant thresholds that now serve as analytical benchmarks (Kim et al. 2012; Ma et al. 2013). Codex Alimentarius specifies maximum melamine levels of 1 mg kg^−1^ in powdered infant formula and 2.5 mg kg^−1^ in other foods, with a lower limit of 0.15 mg kg^−1^ established for liquid infant formula to distinguish unavoidable background contamination from deliberate adulteration (Maleki et al. 2018). Comparable limits have been adopted within the European Union and the United States, anchoring method validation and regulatory acceptance. These targets, together with the prevalence of economically motivated adulteration driven by milk's perishability, price sensitivity, and long supply chains, underscore the need for analytical techniques capable of rapid, sensitive, and matrix‐tolerant detection (Cantor et al. 2014).

Within this framework, Raman spectroscopy has emerged as a particularly attractive tool for milk quality assessment and adulteration detection (Zhang, Shen, et al. 2025). Native milk constituents exhibit characteristic Raman features that enable compositional profiling, whereas many common adulterants and contaminations possess distinct vibrational signatures that can be directly identified (Nedeljković 2019; Silva et al. 2021). The integration of surface‐enhanced Raman spectroscopy (SERS) has further expanded analytical sensitivity, enabling detection of trace‐level contaminants in complex dairy matrices (Tian et al. 2025). In parallel, the increasing adoption of chemometric and sisted data analysis has improved classification accuracy, quantification robustness, and model generalizability, supporting translation from laboratory studies to practical screening applications (He et al. 2019; Ni et al. 2023).

Despite substantial progress in spectroscopic food analysis, the literature on Raman‐based milk authentication remains fragmented. Earlier reviews and methodological studies largely focused on conventional Raman spectroscopy or first‐generation SERS implementations reported before 2015, with limited consideration of recent advances in nano‐engineered substrates, microfluidic and imaging‐based platforms, portable instrumentation, and AI‐driven analytics (He et al. 2019; Silva et al. 2021; Tian et al. 2025). Moreover, a significant proportion of published studies continue to emphasize single‐adulterant detection under simplified laboratory conditions, offering limited insight into multi‐adulterant scenarios, real‐matrix complexity, reproducibility, and regulatory relevance (Silva et al. 2021; Tian et al. 2025). To address these limitations, the present review systematically surveys Raman spectroscopy‐based milk analysis literature published between 2010 and early 2025, with particular emphasis on post‐2015 developments, during which SERS, chemometrics, and ML approaches have rapidly evolved. The literature was compiled from Scopus, Web of Science, PubMed, and Google Scholar, focusing on peer‐reviewed journal articles, authoritative reviews, and regulatory reports related to milk composition, adulteration, and contamination.

In line with the structure of this review, we first examine conventional milk‐quality assessment techniques and their limitations, thereby establishing the analytical rationale for Raman spectroscopy. We then discuss Raman characterization of milk from different animal sources, followed by detailed strategies for detecting chemical and nutritional adulterants, as well as biological contaminants, in liquid milk systems derived from multiple species (including cow, goat, camel, and human milk). Buffalo milk is addressed in a dedicated section owing to its distinct compositional profile, and economic importance, with emphasis on species‐specific Raman and chemometric models, followed by Raman‐based approaches for milk powder analysis. Throughout this review, adulteration refers to the intentional addition or substitution of substances for economic gain, whereas contamination denotes the unintended introduction of biological, chemical, or physical hazards during production, processing, or storage. By integrating spectral chemistry, analytical performance, and regulatory considerations, this review aims to provide a coherent and up‐to‐date framework for advancing Raman‐based milk authentication towards field‐ready, industry‐relevant, and regulator‐aligned applications.

## 2 Conventional and Emerging Approaches for Milk Quality Assessment: Limitations and the Rationale for Raman Spectroscopy

### 2.1 Milk Adulteration Landscape and Conventional Analytical Approaches

Milk is one of the most economically significant and nutritionally complex food commodities, yet it remains highly vulnerable to adulteration, second only to olive oil because of a confluence of drivers: economic pressures, supply–demand imbalance, perishability, fragmented production networks, and uneven regulatory enforcement (Moore et al. 2012). These conditions enable a spectrum of economically motivated adulteration, from dilution with water or whey, through nitrogen addition (e.g., urea and melamine) to inflate apparent protein or solids‐not‐fat values, to the illicit use of preservatives such as formalin to mask spoilage (Azad and Ahmed 2016; Fischer et al. 2011; Singh and Gandhi 2015). The conventional analytical toolbox is broad and, for targeted applications, extremely capable. Foreign proteins from plant sources (e.g., soy, rice, almond, pea, wheat, and lupin) are typically identified using SDS–PAGE, enzyme‐linked immunosorbent assay, high‐performance liquid chromatography (HPLC), near‐infrared (NIR) spectroscopy, and mass spectrometry (MS) (Kolar et al. 1979; Sanchez‐Monge et al. 2004; Scholl et al. 2014). Species‐level adulteration, such as addition of cow milk to goat or buffalo milk, is assessed via inductively coupled plasma‐optical emission spectroscopy, isotope ratio MS, reverse‐phase‐HPLC, HPLC–electrospray ionization–MS, mitochondrial DNA‐based principal component regression, and high‐resolution melting analysis (Bakircioglu et al. 2011; Rosceli Menezes de Oliveira et al. 2016; Song et al. 2011). Highly toxic adulterants, notably melamine and urea, are detected using liquid chromatography–tandem MS, atmospheric pressure chemical ionization–MS, extraction electrospray ionization–MS, Fourier‐transform infrared spectroscopy (FT‐IR) (including attenuated total reflectance), spectrophotometric assays, enzyme‐based sensors, and nanoparticle‐assisted colorimetric methods (Khan et al. 2015; Kim et al. 2012; Moore et al. 2012).

Despite excellent sensitivity and specificity in the laboratory, these techniques entail extensive sample preparation, reagent consumption, high operational costs, and dependence on skilled personnel. Many workflows are destructive or narrowly targeted to known adulterants, limiting their suitability for untargeted screening. Reliance on centralized laboratories further constrains real‐time and on‐site monitoring across complex dairy supply chains. These limitations collectively motivate analytical platforms that combine speed, chemical specificity, minimal sample preparation, and compatibility with intact milk matrices.

### 2.2 Raman Spectroscopy as a Strategic Analytical Platform for Dairy Matrices

Raman spectroscopy directly addresses these needs by providing rapid, label‐free, and nondestructive analysis with molecular‐level specificity (Chandra et al. 2024; Jiang et al. 2025). Its advantage stems from probing intrinsic vibrational modes via inelastic light scattering. In milk, Rayleigh scattering, an elastic process, manifests as turbidity arising from fat globules and casein micelles, whereas fluorescence from riboflavin and aromatic amino acids can overwhelm weak vibrational bands. Raman scattering differs fundamentally: a small fraction of photons exchange energy with molecular vibrations, generating stokes and anti‐stokes shifts that encode direct molecular structure. The resulting molecular fingerprint resolves both native milk constituents and adulterants within complex matrices (Mazurek et al. 2015; Silva et al. 2021). Raman intensity follows polarizability‐driven selection rules and is proportional to *dα*/*dQ* ^2^, where *α* represents the molecular polarizability, the ease with which a molecule's electron cloud can be distorted by an external electric field, and *Q* denotes the normal coordinate of vibration, describing displacement along a vibrational mode (Long 2002). Vibrations that cause large changes in polarizability, such as C─H, C═O, C─C, and N─H stretches, therefore yield strong Raman features. In milk, characteristic bands include proteins (amide I ∼1660 cm^−1^; amide III ∼1240 cm^−1^), lipids (CH_2_ stretching ∼2850–2950 cm^−1^; C═C ∼1650 cm^−1^), and lactose (C─O─C/C─O─H ∼1000–1150 cm^−1^) (He et al. 2019; Mazurek et al. 2015; Surkova and Bogomolov 2023). Adulterants superimpose or perturb these fingerprints, for example, melamine (triazinic ring vibrations ∼680 and ∼980 cm^−1^), and urea (C─N and N─H modes), enabling qualitative recognition and quantitative determination within a single measurement.

### 2.3 Sensitivity Enhancement Through SERS

**FIGURE 1:**
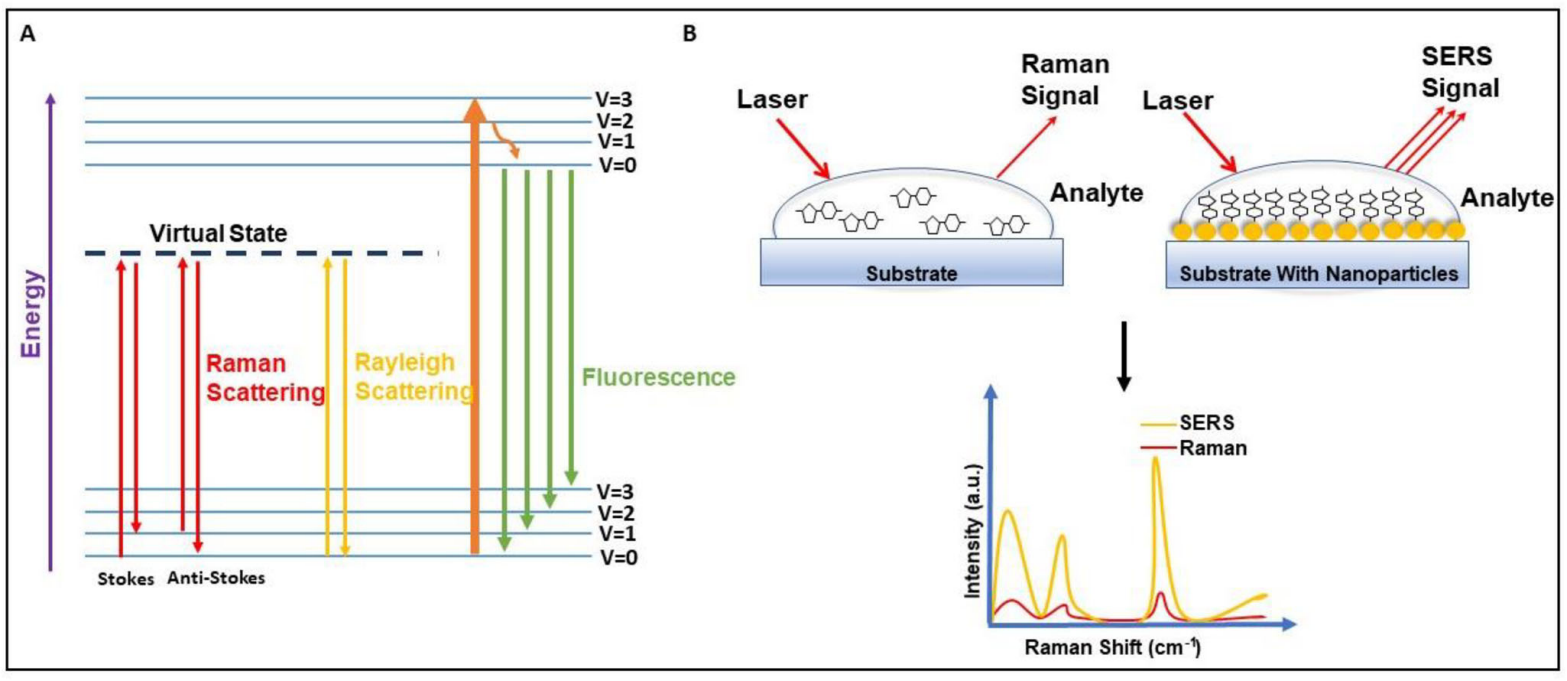
**Photophysical basis of Raman scattering and SERS enhancement**. (A) Energy‐level diagram contrasting Rayleigh scattering, Raman scattering (stokes and anti‐stokes), and fluorescence emission. (B) Schematic of SERS, illustrating localized surface plasmon resonances on metallic nanostructures that amplify Raman signals near adsorbed molecules. SERS, surface‐enhanced Raman spectroscopy.

SERS boosts weak vibrational signals by orders of magnitude through electromagnetic (localized surface plasmon resonance, LSPR) and chemical enhancement. Nanostructured metallic substrates, typically silver, gold, or composites, create intense near‐field regions that amplify Raman scattering from adsorbed molecules. In real‐matrix milk systems, SERS routinely enables trace‐level detection of hazardous adulterants such as melamine, urea, detergents, sucrose, hydrogen peroxide, antibiotics, and aflatoxins, frequently achieving ppm‐to‐ppb ranges (Hussain et al. 2019) (Li, Hussain, et al. 2024) (Li, Wang, et al. 2016). Practically, SERS also mitigates fluorescence and improves spectral resolution, revealing diagnostic bands otherwise obscured in conventional Raman measurements. Substrate functionalization (e.g., small‐molecule ligands, aptamers) further enhances selectivity and supports multiplexed detection. Figure 1 summarizes the photophysical basis of Raman scattering and fluorescence interference (Panel A) and illustrates how nanostructured metallic substrates amplify Raman signals through LSPR in SERS (Panel B).

### 2.4 Analytical Scope Across Animal Milks

Milk from different species exhibits substantial biochemical diversity, which directly influences Raman spectral profiles. Differences in casein‐to‐whey ratios, lipid chain length and unsaturation, and carbohydrate composition manifest as band position shifts, intensity ratio changes, and baseline behavior. For example, human milk often presents stronger whey‐associated amide bands and unsaturated lipid signatures, whereas cow, goat, and buffalo milks display dominant casein features and more saturated lipid markers. Camel milk can exhibit distinctive carbohydrate/immunoglobulin‐related bands, whereas donkey and yak milks reveal unique lipid unsaturation signatures reflecting physiology and environment (Andreas et al. 2015; Swelum et al. 2021; Balthazar et al. 2017; Eidelman and Schanler 2012; Kumar et al. 2014; Wang et al. 2023; Zicarelli 2004). Beyond species composition, biological factors, lactation stage, diet, and animal health modulate spectral characteristics. Colostrum and early lactation milk typically exhibit enhanced protein‐ and lipid‐associated peaks; dietary fat sources influence CH stretching and C═C vibrational intensities. Processing steps (pasteurization, homogenization, and drying) may induce subtle conformational changes, apparent in band sharpness and baseline shifts, without compromising chemical specificity (Nedeljković 2019; Silva et al. 2021). From an authentication standpoint, these interspecies and processing‐driven variations enable species substitution and compositional fraud detection. Although closely related species can share overlapping features, chemometric modeling and, where needed, SERS enhancement provide robust discrimination grounded in molecular structure rather than bulk physicochemical properties (Tian et al. 2025). Detailed species‐specific markers and human milk applications (macronutrient profiling; emerging contaminants) are addressed in Section 3.

### 2.5 Comparative Overview of Raman‐Based Approaches for Milk Authentication

To position Raman spectroscopy within realistic deployment scenarios, Table 1 provides a comparative overview of the principal Raman modalities applied to milk authentication, summarizing their detectable adulterants, typical sensitivity ranges, key advantages, limitations, and optimal use cases. Conventional Raman spectroscopy is well suited for general milk profiling, enabling rapid and direct assessment of major compositional markers such as proteins, fats, and lactose. However, fluorescence originating from native milk chromophores can restrict sensitivity, particularly when targeting low‐level adulterants in complex matrices. SERS, typically Ag/Au substrates, offers ultra‐trace detection capability (ppm–ppb) for a wide range of toxic adulterants, including melamine, urea, ammonium sulfate (AmS), antibiotics, and even engineered materials (e.g., carbon nanotubes [CNTs]). Despite its outstanding sensitivity, SERS performance is influenced by substrate reproducibility and competition from matrix components for surface adsorption. These challenges can be substantially alleviated through advances in surface engineering and careful protocol optimization (Hussain et al. 2019; Li, Hussain, et al. 2024; Li, Wang, et al. 2016).

**TABLE 1:** Comparative analytical performance of Raman‐based techniques for detection of milk adulterants and contaminants.

Raman modality	Target adulterants/contaminants	Typical LOD range	Detection mechanism	Key strengths	Major constraints	Application readiness
Conventional Raman	Major milk constituents, gross adulteration	High ppm–%	Intrinsic molecular vibrational scattering	Rapid, minimal sample prep	Fluorescence, low sensitivity	Laboratory screening
SERS (Ag/Au)	Toxic adulterants, antibiotics, residues	Low ppm–ppb	Plasmonic enhancement	Ultra‐trace sensitivity	Substrate reproducibility, matrix effects	Advanced lab /regulatory
Microfluidic Raman	Antibiotics, small polar adulterants	ppb (with SERS)	Miniaturized confinement, and enhancement	Low sample volume, fast	Channel fouling; fabrication complexity	Prototype/translational
FT‐Raman	Powdered adulterants	Mid‐ppm	NIR excitation suppresses fluorescence	Suitable for dry matrices	Poor aqueous sensitivity	Milk powder analysis
Hyperspectral Raman	Multiple adulterant mixtures	≥0.05%	Spatial–spectral imaging	Multi‐adulterant discrimination	Cost, slow acquisition	Research/forensic
Portable Raman	Common chemical adulterants	ppm	Handheld vibrational sensing	Field deployable	Lower spectral resolution	On‐site screening

Microfluidic Raman platforms integrate Raman detection with on‐chip sample handling, and when combined with SERS, enable ppb‐level detection of antibiotics, melamine, and thiocyanate (SCN). The approach offers notable advantages in terms of speed, reduced sample volume, and portability. Practical limitations include microchannel clogging and surface fouling, particularly under high fat or particulate loads; these effects can be mitigated through appropriate channel design and pre‐filtration strategies. FT Raman spectroscopy, employing NIR excitation, significantly suppresses autofluorescence and is therefore advantageous for dry or powdered milk matrices, such as whey and starch adulterants, achieving sensitivities in the mid‐ppm range. Owing to the absence of signal enhancement, FT Raman is less suitable for trace‐level regulatory detection but remains effective for screening applications and compositional verification (de Almeida et al. 2012; Mazurek et al. 2015). Hyperspectral Raman spectroscopy extends conventional Raman analysis by incorporating spatial mapping and chemical imaging, facilitating the detection and visualization of multiple adulterants, including melamine, urea, and dicyandiamide (DCD) at concentration levels of approximately 0.05%–0.1%. Its ability to reveal spatial heterogeneity within samples makes it particularly valuable for multi‐adulterant mapping and method development, although high instrumentation costs and longer acquisition times currently limit routine deployment (Wang et al. 2017). Portable (handheld) Raman instruments enable on‐site screening with typical detection limits in the ppm range for common adulterants such as melamine, urea, and SCN. Although issues related to spectral resolution, accuracy, and calibration transfer must be carefully managed, the gains in mobility and rapid decision‐making are substantial, especially at milk collection centers and along supply‐chain logistics nodes (Mecker et al. 2012; Nieuwoudt et al. 2017). Having established the rationale, analytical performance, and practical scope of Raman spectroscopy and its variants for milk authentication, Section 3 focuses on species‐specific Raman signatures and applications to human breast milk, with particular emphasis on compositional markers, macronutrient profiling, and the detection of emerging adulterants and contaminants.

## 3 Raman Spectroscopy for Milk Analysis: Species‐Specific Signatures and Human Breast Milk Applications

Raman spectroscopy has emerged as a powerful, noninvasive analytical tool for characterizing milk across species, offering chemically resolved insights into composition and structure. Although the number of studies remains comparatively limited, existing evidence demonstrates its ability to probe macronutrient profiles, lipid organization, and emerging contaminants in complex biological matrices, capabilities that conventional assays often lack.

### 3.1 Species‐Specific Raman Signatures of Milk

Milk from different animal species fulfills diverse nutritional, physiological, and therapeutic roles, and these functional differences are mirrored in molecular composition (Elrefaey and Eissa 2025). Variations in protein secondary structure, lipid chain length and unsaturation, and carbohydrate content produce reproducible differences in Raman band positions and intensity ratios (He et al. 2019). Biological factors such as lactation stage, diet, and health status introduce additional variability that must be considered during interpretation. From an analytical perspective, Raman spectroscopy excels at resolving these interspecies differences because it probes intrinsic molecular vibrations rather than bulk physicochemical properties (He et al. 2019; Silva et al. 2021). Differences in casein‐to‐whey ratios, lipid saturation profiles, and lactose content manifest as changes in the amide I and III regions, CH stretching modes, and carbohydrate‐associated bands. When combined with normalization strategies and multivariate analysis, these spectral features enable reliable discrimination between milk types and underpin detection of species‐based adulteration, such as substitution of high‐value milk with cow milk (Meurens et al. 2005; Saleem et al. 2021; Ullah et al. 2017). Microstructural attributes further influence Raman measurements. Species‐dependent differences in fat globule size, casein micelle organization, and aqueous‐phase composition affect scattering behavior and baseline characteristics. Processing steps, including pasteurization, homogenization, and drying, can induce subtle conformational changes, altering band sharpness and baseline without compromising chemical specificity. Understanding these effects is essential for robust interpretation of Raman data from raw, processed, and powdered milk (Hussain et al. 2019; Tan and Chen 2016).

### 3.2 Human Breast Milk Analysis

Human breast milk represents a uniquely complex and dynamic biological system, tailored to infant development. Beyond balanced nutrition, it delivers immunological protection, bioactive molecules, and developmental cues. Raman spectroscopy offers a nondestructive platform for probing this complexity, supporting both nutritional assessment and contaminant surveillance.

#### 3.2.1 Macronutrient Profiling and Quantification

**FIGURE 2:**
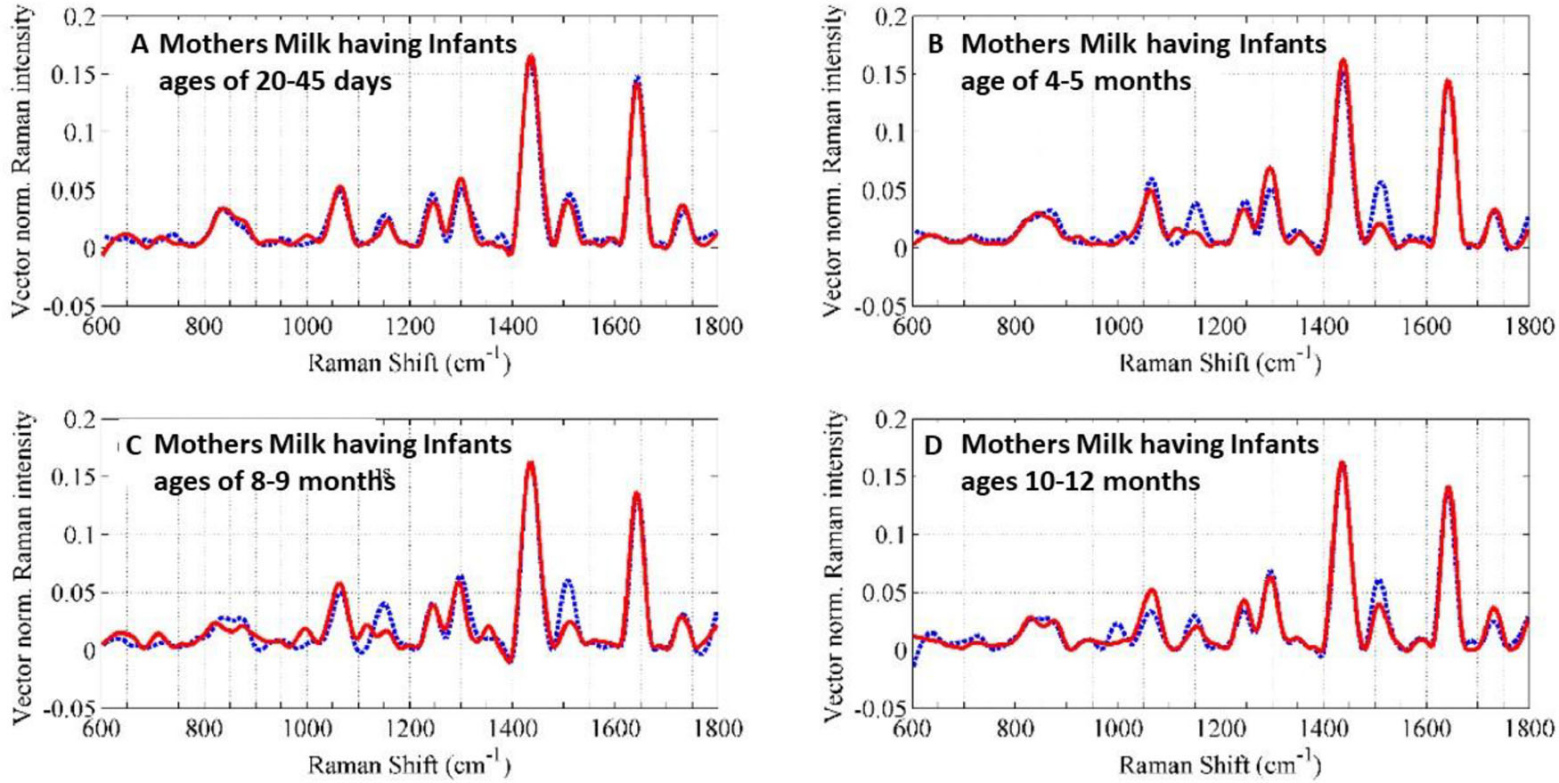
**Sex‐specific Raman spectral profiles of human breast milk**. Comparison of Raman spectra from breast milk samples collected at different lactation stages, illustrating compositional differences linked to infant sex.The spectra correspond to milk samples from mothers nursing infants aged (A) 20–45 days, (B) 4–5 months, (C) 8–9 months, and (D) 10–12 months. The dotted blue line represents milk from mothers nursing male infants, characterized by higher carotenoid and saccharide signals, whereas the solid red line corresponds to milk for female infants, showing elevated fatty acids, phospholipids, and tryptophan. These spectral variations highlight the potential of Raman spectroscopy for sex‐specific nutritional assessment. *Source*: Figure adapted from Ullah et al. 2018, with permission from Optica.

Accurate macronutrient quantification in human milk is critical for ensuring adequate energy, protein, and fat intake, particularly for premature or low‐birth‐weight infants, and underpins individualized fortification strategies and milk‐bank quality control (Pereira‐da‐Silva and Cardoso 2025). Motta et al. (2012) demonstrated that NIR Raman spectroscopy enables rapid, nondestructive quantification of human milk composition, with spectra dominated by casein, lactose, and fatty‐acid‐associated bands within the 600–1800 cm^−1^ region. Their study revealed stable lactose levels across feeding periods, accompanied by increasing lipid content, consistent with physiological changes from foremilk to hindmilk. A carotenoid band near 1520 cm^−1^ highlighted sensitivity to maternal diet (Figure 2). While promising, limitations included spectral overlap among lipid components, model residuals, and a small sample cohort. Future improvements will require larger datasets, refined biochemical modeling, and portable Raman systems integrated with chemometric or ML tools for real‐time nutritional classification.

Beyond composition, Raman spectroscopy has been explored for differentiating breast milk by infant sex, reflecting sex‐specific growth trajectories. Ullah et al. (2018) combined Raman spectroscopy with support vector machine (SVM) modeling to classify milk produced for male versus female infants, achieving 86% accuracy based on discriminatory bands between 838 and 1730 cm^−1^. Female‐infant milk exhibited higher fatty acids, phospholipids, and tryptophan signals, whereas male‐infant milk showed elevated carotenoids and saccharides. These compositional differences are clearly illustrated in Figure 2, which compares Raman spectra of breast milk from mothers nursing male and female infants. However, the modest sample size (48 samples) limits statistical robustness, underscoring the need for multicenter cohorts and advanced classifiers to validate these observations across populations and lactation stages.

**FIGURE 3:**
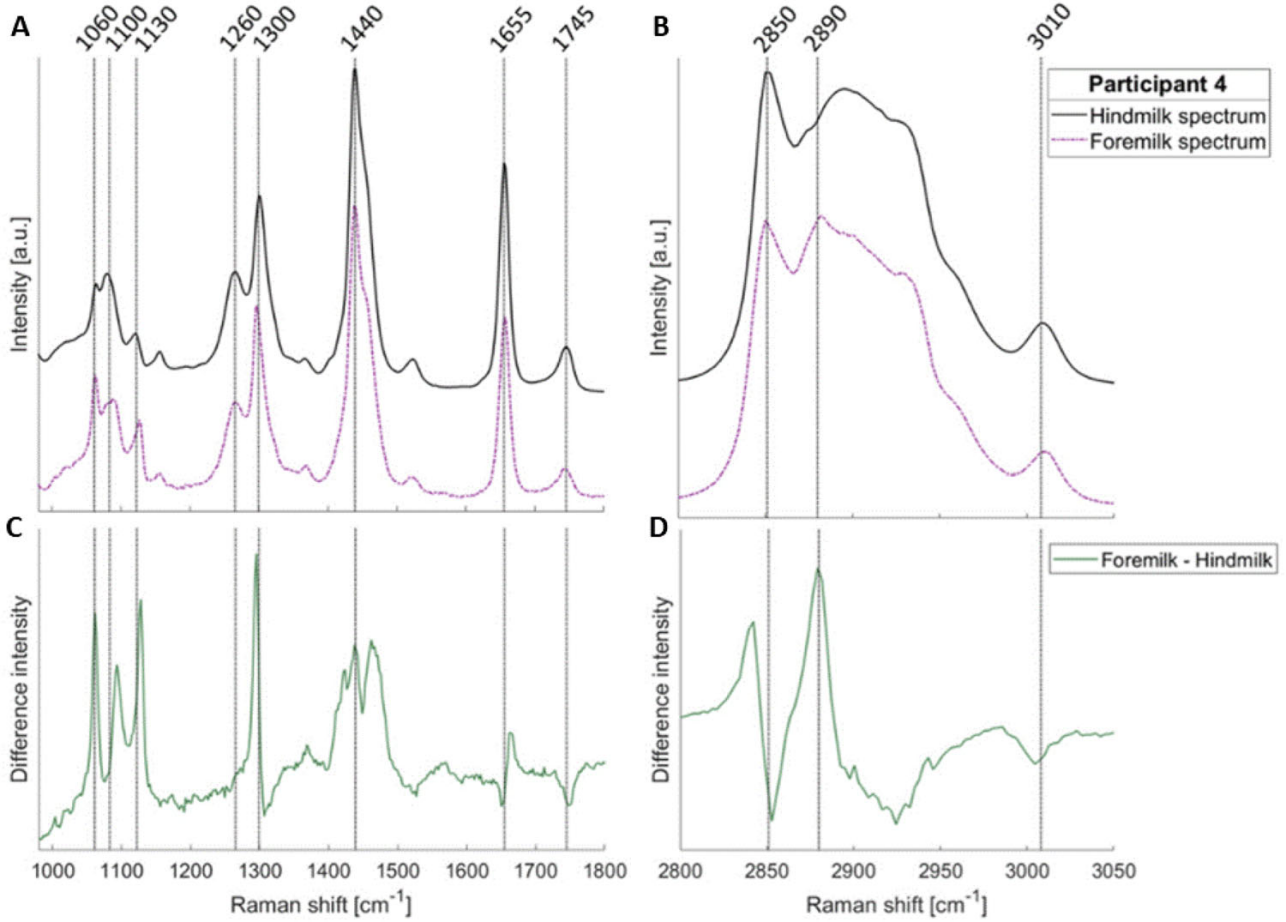
**Lipid‐phase transitions in human breast milk: Raman spectral differences between foremilk and hindmilk**. Representative Raman spectra from foremilk and hindmilk collected from one of the donors, illustrating structural changes in lipid organization. Panels (A) and (B) show the fingerprint and high‐wavenumber regions, respectively, whereas Panels (C) and (D) present the corresponding difference spectra. Foremilk exhibits sharp lipid‐associated bands at ∼1060 and ∼1130 cm^−1^ and a well‐defined peak near 2890 cm^−1^, characteristic of fatty acids in a more ordered, crystalline phase with higher trans conformations. Hindmilk displays broader features and shifts in the ∼1300 and ∼1440 cm^−1^ regions, indicating increased conformational disorder and a predominantly liquid‐like lipid phase. *Source*: Figure adapted from de Wolf et al. 2021 with permission from Optica.

High‐resolution Raman imaging has further expanded insight into lipid organization within human milk. Using confocal Raman microscopy, de Wolf et al. compared foremilk and hindmilk during breastfeeding, revealing a transition from near‐crystalline to more liquid‐like lipid phases. Foremilk exhibited sharp lipid‐associated bands at ∼1060 and ∼1130 cm^−1^ and a well‐defined peak near 2890 cm^−1^, indicative of ordered lipid packing, whereas hindmilk showed broader features and shifts in the ∼1300 and ∼1440 cm^−1^ regions, consistent with increased conformational disorder (Figure 3). Additional changes at 1260, 1655, and 3010 cm^−1^ further confirmed structural reorganization. These findings highlight dynamic lipid‐phase behavior with potential nutritional implications. Although fatty‐acid chain length and unsaturation remained stable, structural reorganization was evident. Limitations included small sample numbers and room‐temperature measurements, underscoring the need for future work incorporating temperature control, in vivo acquisition, and advanced spectral deconvolution (de Wolf et al. 2021).

#### 3.2.2 Detection of Microplastics

Microplastic contamination in human breast milk has emerged as a critical public health concern, as particles can enter the maternal body via food, water, and environmental exposure and may transfer to infants during breastfeeding. Raman spectroscopy is particularly suited to microplastic identification because it provides polymer‐specific vibrational fingerprints without chemical labeling (Nadarasan et al. 2025). Ragusa et al. (2022) detected microplastics in 26 of 34 breast milk samples, with particles appearing as irregular fragments or spheres ranging from 2 to 12 µm. Dominant polymers included polyethylene, polyvinyl chloride, and polypropylene, each confirmed by characteristic Raman bands 2022. No clear association with maternal demographic or lifestyle factors was observed. Limitations included small sample volumes and challenges in detecting very small particles. Future research will require larger cohorts, high‐resolution Raman instrumentation, automated spectral classification, and systematic assessment of infant health implications.

#### 3.2.3 Detection of Antibiotic Residues

Monitoring antibiotic residues in human milk is essential to protect infant health, as even trace exposure may disrupt gut microbiota, provoke allergic reactions, or contribute to antimicrobial resistance. Raman spectroscopy, particularly when coupled with SERS and ML, offers a promising route for ultra‐trace detection in complex biological matrices. Mou et al. (2024) employed label‐free SERS integrated with a pseudo‐Siamese deep‐learning network for simultaneous detection of tetracycline‐class antibiotics in human milk 2024 Distinct vibrational features enabled discrimination between doxycycline and tetracycline, even in mixed samples, with a detection limit of 10^−9^ M and strong repeatability. Despite these advances, challenges remain related to substrate stability, spectral overlap, and limited reference libraries. Continued development of robust SERS substrates, expanded antibiotic databases, and portable platforms will be critical for translating these approaches into routine milk‐bank and clinical screening.

Collectively, these studies underscore Raman spectroscopy's versatility in resolving species‐level compositional differences and human milk complexity while extending its scope to emerging contaminants and trace‐level pharmaceutical residues. Beyond nutritional profiling, Raman spectroscopy's sensitivity to subtle molecular perturbations arising from processing, physiological variability, and low‐level contamination positions it as a cornerstone for future authenticity assurance and health‐risk monitoring. Building on these insights, Section 4 examines Raman‐based strategies for detecting adulteration in liquid milk, focusing on methodological advances, performance metrics, and real‐world applicability. Table 2 summarizes representative Raman and SERS spectral markers reported for human breast milk, covering macronutrients, micronutrients, lipid‐associated bands, nucleotides, hormones, and selected antibiotic residues. These diagnostic vibrational features underpin nutritional profiling, sex‐specific classification, and contaminant detection using both benchtop and portable Raman platforms.

**TABLE 2:** Raman and SERS spectral markers reported for human breast milk.

(A) Macronutrients and micronutrients (785 or 830 nm excitation, 350 mW) (Motta et al. 2012)	
Raman/SERS band (cm^−1^)	Assignment (functional group/molecule)
1200–1800	Lipids, proteins, carbohydrates (composite region)
1650	Amide I band (proteins, predominantly casein)
1440	CH_2_ bending (lipids, fatty acids, triolein)
1060–1080	C─O/C─C stretching (lactose, carbohydrates)
1006	Carotenoids—ring breathing/C─C stretching
1154	Carotenoids—C─C stretching (polyene chain)
1528	Carotenoids─C═C stretching (conjugated polyene chain)

(B) Sex‐specific and compositional markers (μRamboss, DONGWOO OPRTON; CW excitation at 532 nm) (Ullah et al. 2018)	
Raman/SERS band (cm^−1^)	Assignment
838	Saccharides (polysaccharides)
1120	Saccharides
1150	Carotenoids─C─C stretching
1515	Carotenoids─C═C stretching
1065	Triolein/oleic acid/palmitic acid (fatty acids, cholesterol‐related)
1440	CH_2_ bending of fatty acids
1640	Amino acids—tryptophan (Trp)
1250	Cytosine (nucleotide)
1290–1296	Phospholipids (choline‐, inositol‐, serine‐containing)
1730–1732	C═O stretching vibration of cortisone

(C) Antibiotic residues detected by portable SERS using Anton Paar Cora100 Raman spectrometer (*λ* = 785 nm, 25 mW) (Mou et al. 2024)	
Raman/SERS band (cm^−1^)	Assignment
1316	Rocking vibration of C1–H33 and C8–H34
1628	Symmetric stretching of C21–C28 and bending of C29–O32–H57
1218	Characteristic doxycycline vibrational mode
1268	Amide triple–doublet vibration
1616	C═O stretching vibration
1238	Characteristic tetracycline vibrational mode
1314	Characteristic tetracycline vibrational mode

## 4 Raman‐Based Strategies for the Detection of Adulteration in Liquid Milk

Raman spectroscopy has become an indispensable analytical platform for detecting adulteration in liquid milk owing to its molecular specificity, minimal sample preparation, and compatibility with complex aqueous matrices (Li et al. 2022; Ullah et al. 2017). Unlike conventional compositional assays that rely on bulk physicochemical parameters, Raman‐based approaches interrogate intrinsic vibrational signatures of milk constituents, enabling detection of both subtle compositional manipulation and trace‐level contaminants (Nedeljković 2019). When coupled with chemometric modeling and SERS, these methods provide robust discrimination across a wide spectrum of adulteration practices encountered in modern dairy supply chains (Tian et al. 2025). This section reviews Raman‐based strategies for liquid milk adulteration detection, categorized according to species substitution, chemical adulterants, nutritional manipulation, biological contamination, and emerging unconventional adulterants.

### 4.1 Species Adulteration

Species adulteration involves the intentional substitution or dilution of milk from one animal source with that from another, typically motivated by economic gain or supply limitations. Such practices compromise authenticity, nutritional labeling, and consumer trust, and may pose risks for individuals with specific dietary or allergenic sensitivities. Raman spectroscopy enables species‐level discrimination by capturing differences in protein composition, lipid structure, and carbohydrate profiles arising from inherent biochemical variation between animal milks.

#### 4.1.1 Detection of Goat Milk in Cow Milk

The adulteration of cow milk with goat milk, or vice versa, may occur deliberately to modify yield or perceived nutritional value, or unintentionally through cross‐contamination during collection and processing (Li et al. 2022; Song et al. 2011). Discrimination is challenging because of the high compositional similarity between the two milk types. Li et al. (2022) investigated goat milk adulteration in cow milk using Raman spectroscopy combined with principal component analysis (PCA) and partial least squares regression (PLSR). Raman spectra of pure cow milk, goat milk, and their mixtures exhibited highly overlapping profiles within the 715–1812 cm^−1^ range, dominated by fat‐ and protein‐related bands. Key spectral features included 1004 cm^−1^ (phenylalanine ring breathing), 1064–1081 and 1120 cm^−1^ (C─C stretching of fatty acids), 1304 cm^−1^ (CH_2_ twisting), 1441 cm^−1^ (CH_2_ scissoring), 1656 cm^−1^ (amide I and C═C stretching), and 1750 cm^−1^ (ester carbonyl stretching). These similarities rendered visual discrimination ineffective. However, chemometric modeling enabled reliable quantification of adulteration, achieving an *R* ^2^ of 0.9781 with a root mean square error of prediction (RMSEP) of 3.82%. Multi‐point spectral averaging reduced sample inhomogeneity effects arising from fat globule distribution (Li et al. 2022).

The authors highlighted the potential of advanced preprocessing, hyperspectral Raman mapping, SERS enhancement, and integration with deep‐learning algorithms to improve robustness and sensitivity for routine industrial deployment. Complementing this work, Zhang et al. (2025) applied Raman spectroscopy coupled with Gaussian‐weighted *k*‐nearest neighbor regression to quantify cow milk adulteration in goat milk. The proposed model outperformed ridge regression, LASSO, and support vector regression, achieving an *R* ^2^ of 0.962 and a mean squared error of 0.005. Discriminatory features were primarily located in the 850–870 cm^−1^ (C─C stretching), 1050–1120 cm^−1^ (C─N and P═O stretching), and 1640–1670 cm^−1^ (amide I) regions. Although prediction accuracy was high, performance remained sensitive to spectral preprocessing and environmental noise, underscoring the need for standardized analytical pipelines and calibration transfer strategies. The study further emphasized the promise of portable Raman systems for real‐time, on‐site authentication in dairy processing environments (Zhang, Shen, et al. 2025).

### 4.2 Chemical Adulterants

Common adulterants, such as melamine, SCN, urea, and related nitrogen‐rich compounds, have been intentionally added to milk to falsify protein content or extend shelf life. This section further discusses the Raman and SERS‐based detection strategies employed for identifying these adulterants, highlighting their characteristic spectral fingerprints and analytical performance in milk matrices.

#### 4.2.1 Melamine

Melamine has been deliberately added to milk and dairy products to artificially inflate apparent protein content during nitrogen‐based quality assessment, enabling dilution with water while still meeting regulatory thresholds and maximizing economic gain (Kim et al. 2012; Li, Yang, et al. 2016). This practice poses severe public health risks, as melamine ingestion is associated with kidney stone formation, nephrotoxicity, renal failure, and increased mortality, with infants and young children particularly vulnerable. These hazards have driven sustained interest in analytical techniques capable of detecting melamine rapidly, sensitively, and reliably within complex dairy matrices. Raman spectroscopy, and especially SERS, has emerged as a promising approach for melamine detection owing to its molecular specificity, rapid acquisition, and minimal sample preparation (Li, Yang, et al. 2016). However, the intrinsic complexity of milk presents significant analytical challenges. Proteins, lipids, and lactose compete for adsorption sites on plasmonic substrates and generate strong background scattering, which can severely suppress or obscure melamine's characteristic Raman signatures.

**FIGURE 4:**
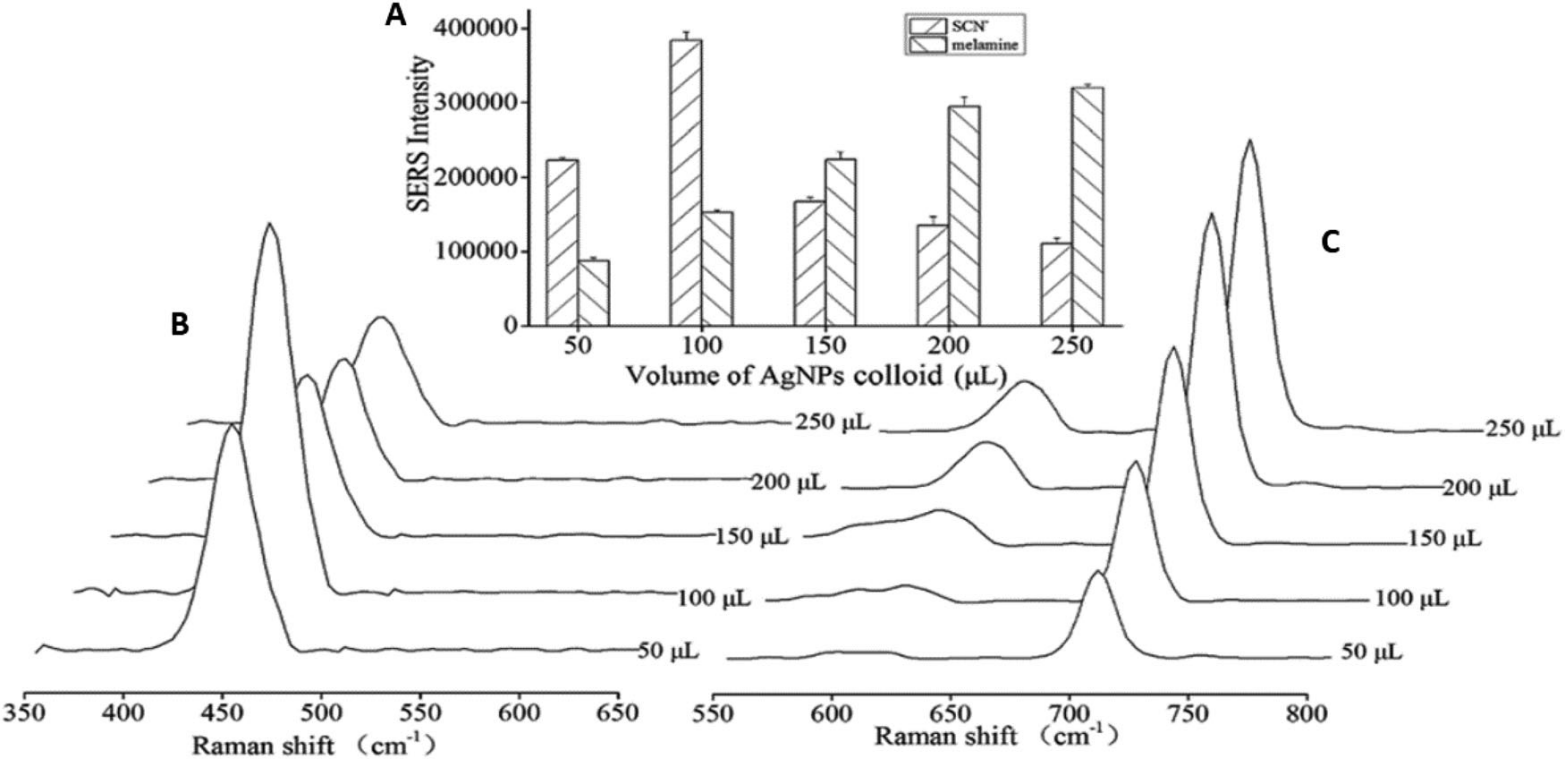
**Influence of AgNP colloid volume on SERS signal intensities of SCN** **and melamine**: Effect of AgNP colloid volume on SERS responses for SCN and melamine under optimized experimental conditions (20 mL of 0.2 mol L^−1^ NaCl, 200 µL standard solution, 250 µL of 2.0 mol L^−1^ NaCl, and 400 µL of 1.0 mol L^−1^ NaOH; mixing times: 0 min for SCN and 6 min for melamine). Melamine intensity increased steadily with colloid volume, whereas SCN showed a nonlinear trend, rising initially and then declining, indicating stronger dependence on colloid concentration. Based on these trends, 100 mL AgNP colloid was selected for subsequent experiments. Panels: (A) processed data as column chart; (B) SERS spectra of SCN; (C) SERS spectra of melamine. *Source*: Figure adapted from Yang et al. 2014 with permission from The Royal Society of Chemistry.

Yang et al. (2014) systematically investigated matrix interference in SERS‐based detection of melamine and SCN in liquid milk and milk powder using silver nanoparticle (AgNP) substrates. In undiluted milk, melamine signals at approximately 700 and 915 cm^−1^ were completely masked due to competitive adsorption and enhanced optical scattering. Detectable signals only appeared after dilution exceeding threefold, yet even at dilution factors as high as 75×, peak intensities remained substantially lower than those observed in aqueous solutions. Consequently, the limit of detection (LOD) increased dramatically from 2.5 ppb in water to approximately 2 ppm in milk. Additional attenuation occurred after sample drying, attributed to substrate fouling and surface blockage. These findings underscored matrix interference as a major limitation of conventional SERS approaches. To optimize detection, Yang et al. evaluated the effect of AgNP colloid volume on SERS responses for SCN and melamine (Figure 4). Panel A shows a column chart of processed data, whereas Panels B and C present representative SERS spectra for SCN and melamine, respectively. The results revealed contrasting trends: melamine intensity increased monotonically with colloid volume, whereas SCN exhibited an initial rise followed by a decline, indicating a stronger dependence on colloid concentration. On the basis of these observations, an AgNP colloid volume of 100 mL was selected for subsequent experiments. This optimization step highlights the importance of controlling nanoparticle concentration to balance signal enhancement and matrix suppression (Yang et al. 2014).

Significant progress in addressing substrate stability and reproducibility was reported by Li et al. (2016), who employed silica‐coated gold nanoparticles (Au@SiO_2_) for melamine detection in milk. The silica shell provided controlled nanoparticle aggregation, reduced nonspecific adsorption, and enabled the formation of stable plasmonic hot spots, resulting in superior spectral reproducibility compared with bare gold nanoparticles (AuNPs). Distinct melamine bands at 380, 581, 676, and 984 cm^−1^ were consistently observed, with the 676 cm^−1^ triazine‐ring vibration used for quantitative evaluation. The method exhibited linearity over the concentration range of 0.5–5 mg L^−1^ (*R* ^2^ = 0.9895), achieved a detection limit below 1 mg L^−1^, and demonstrated recoveries of 94.6%–102.5% in spiked milk samples. Despite these advances, challenges associated with matrix variability and controlled nanoparticle aggregation remain. Future directions highlighted include the integration of portable SERS instrumentation, automated chemometric modeling, and rational substrate design aimed at achieving ultra‐trace detection under realistic dairy processing conditions (Li, Yang, et al. 2016).

Alternative Raman‐based strategies have also been explored. Tan and Chen (2016) investigated coffee‐ring‐assisted Raman spectroscopy for qualitative melamine detection. The coffee‐ring effect refers to the phenomenon where solutes in a drying droplet migrate to the perimeter, forming a concentrated ring that enhances local analyte density and signal intensity. In this study, gold‐coated substrates produced well‐defined ring morphologies and reduced fluorescence backgrounds compared with aluminum, stainless steel, and molybdenum surfaces. A diagnostic melamine peak at approximately 691 cm^−1^ enabled detection down to ∼1 ppm when combined with discrete wavelet transform processing. However, quantitative reproducibility was limited by uneven analyte distribution, surface hydrophobicity effects, and sensitivity to fluorescence interference. The authors noted that coupling coffee‐ring deposition with SERS‐active substrates, along with automated spectral mapping and ML‐assisted interpretation, could substantially improve robustness (Tan and Chen 2016).

More recently, Liu et al. (2025) reported a data‐driven Raman approach employing spatiotemporal attention networks for the detection of milk adulteration, including melamine, urea, and water. The model effectively captured both spectral and contextual dependencies within Raman datasets, resolving vibrational features associated with native milk constituents such as proteins (amide I and III), lipids (C─H stretching and C═C vibrations), and carbohydrates (C─O─C and C─O─H modes), alongside adulterant‐specific signatures such as melamine triazine‐ring vibrations. When evaluated across a large dataset of 3000 milk samples using 10‐fold cross‐validation, the spatiotemporal attention networks framework achieved an accuracy of 94.6% and an *F*1 score of 93.0, outperforming conventional ML and deep‐learning architectures including convolutional neural network (CNN), SVM, random forest, attention‐enhanced recurrent neural networks, hybrid broad deep learning, and YOLOv5l. Although the approach demonstrated strong robustness and convergence efficiency, limitations included computational complexity, reliance on high‐performance hardware, and reduced suitability for real‐time deployment using image‐oriented models. Future research directions include optimization of attention mechanisms for long‐range spectral dependencies, expansion to under‐reported adulterants, improved model interpretability, and integration with portable Raman platforms for real‐time industrial and regulatory monitoring (Liu et al. 2025). Overall, Raman‐ and SERS‐based strategies for melamine detection have evolved from proof‐of‐concept demonstrations to increasingly sophisticated analytical frameworks capable of addressing complex milk matrices. Continued progress in substrate engineering, data analytics, and portable instrumentation is expected to further align Raman‐based methods with regulatory requirements and real‐world dairy quality surveillance. In this context, Table 3 provides a consolidated overview of Raman and SERS strategies for detecting melamine in milk matrices. Section  (A) summarizes the principal diagnostic bands and their vibrational assignments, emphasizing the triazine ring breathing modes and associated N─C─N deformations that underpin melamine's spectral identity. Section  (B) illustrates representative experimental implementations, detailing the platforms, excitation conditions, and configurations employed to capture these characteristic bands in real‐world milk analysis. Collectively, these data demonstrate the robustness and reproducibility of Raman‐based approaches, reinforcing their translational potential for rapid and reliable melamine detection in complex dairy systems.

**TABLE 3:** Raman‐ and sSERS‐based detection of melamine in milk: excitation conditions, enhancement strategies, and analytical performance.

(A) Key diagnostic Raman and SERS bands of melamine	
Raman/SERS band (cm^−1^)	Vibrational assignment
670–690	Triazine ring breathing and N─C─N deformation
381	C─N bending vibration
584	NH_2_ twisting vibration
980–987	Ring breathing I mode (C─N─C/N─C─N bending)
983–984	Characteristic melamine band (triazine ring)
1003	Symmetric N─C─N stretching

(B) Representative Raman and SERS implementations for melamine detection	
Raman/SERS band(s) (cm^−1^)	Instrument/experimental context
679/704	SERS using AgNPs with a portable Raman spectrometer (*λ* = 785 nm, *P* = 150 mW, *t*_exp = 10 s) (Yang et al. 2014)
381, 584, 676, 984	SERS using cyclodextrin modified –AgNPs with a Raman spectrometer (*λ* = 785 nm) (Ma et al. 2013)
672/700; 980/915	Cylindrical SERS substrate with HR 320 Raman system (*λ* = 632.8 nm, *P* = 35 mW) (Rajapandiyan et al. 2015)
984, 1003	SERS using Au@SiO_2_ nanoparticles with a confocal Raman spectrometer (*λ* = 632.8 nm, *P* = 20 mW, *t*_exp = 10 s) (Li, Yang, et al. 2016)
671–691	Confocal Raman spectroscopy using a Renishaw confocal Raman microscope (*λ* = 785 nm) (Tan and Chen 2016)
983	Line‐scan Raman chemical imaging system (*λ* = 785 nm) (Qin et al. 2017)
683, 986	Confocal Raman spectroscopy using Renishaw system 1000 (*λ* = 785 nm) (Nieuwoudt et al. 2016a)

*Note*: λ denotes excitation wavelength; P, laser power; t_exp, exposure time.

#### 4.2.2 Urea

Urea is illicitly added to milk to artificially increase its solids‐not‐fat content, thereby improving apparent texture and taste while reducing production costs. Despite its low cost and ease of addition, excessive urea intake poses serious health risks, including renal dysfunction, gastrointestinal disturbances, metabolic imbalance, and systemic toxicity (Khan et al. 2015). These risks are particularly pronounced in infants, children, and individuals with compromised kidney function, making reliable detection of urea in milk a critical food safety priority (Hussain et al. 2019; Shalileh et al. 2023).

Early Raman‐based investigations demonstrated that urea exhibits distinct vibrational features that can be exploited for quantitative analysis, despite strong background contributions from milk constituents. Khan et al. (2015) evaluated NIR spectroscopy for the quantitative determination of urea in milk without prior sample preparation and used complementary Raman measurements to confirm urea‐specific spectral signatures. The most prominent Raman marker for urea appeared at approximately 1003 cm^−1^, corresponding to the symmetric stretching vibration of the N─C─N moiety. Additional weaker bands were observed at 1170, 1461, 1532, 1570, and 1636 cm^−1^. In contrast, native milk spectra were dominated by intrinsic bands at 1068 and 1124 cm^−1^ (lactose and minerals), 1260 and 1300 cm^−1^ (lipids and proteins), 1440 cm^−1^ (CH_2_ deformation), and 1650 cm^−1^ (amide I and unsaturated lipids) (Khan et al. 2015).

Quantitative analysis revealed a concentration‐dependent increase in the intensity of the 1003 cm^−1^ band, enabling chemometric modeling for urea determination. PLSR outperformed inverse least squares, achieving a coefficient of determination close to 0.99 across a concentration range of 10–1000 mg dL^−1^. However, analytical sensitivity declined below approximately 50 mg dL^−1^ due to reduced signal‐to‐noise ratios and spectral overlap arising from milk turbidity and fluorescence. The authors highlighted the need for larger, more diverse datasets, improved baseline and scattering correction, and advanced enhancement strategies to extend detection limits and support real‐time quality monitoring in industrial settings (Khan et al. 2015). To overcome these sensitivity constraints, Hussain et al. (2019) reported a SERS approach combined with the coffee‐ring effect for the simultaneous detection of urea and AmS in milk. The study employed both AuNPs and gold–silver core–shell nanoparticles (Au@AgNPs), with UV–visible spectroscopy confirming plasmon resonance peaks at 526 nm for AuNPs and dual peaks at 402 and 482 nm for Au@AgNPs. Transmission electron microscopy revealed well‐defined nanostructures consisting of ∼26 nm gold cores coated with ∼6.5 nm silver shells. Raman analysis showed characteristic urea bands at approximately 1001 and 1461 cm^−1^, and Au@AgNPs produced sharper and more intense signals than AuNPs, reflecting stronger electromagnetic enhancement 2019.

This SERS‐based strategy enabled detection of urea down to 5 mg dL^−1^ with excellent linearity (*R* ^2^ ≈ 0.98–0.99), demonstrating its capability for sensitive, dual‐adulterant quantification with minimal sample preparation. Nevertheless, practical limitations remained, including matrix interference, reduced sensitivity near regulatory threshold levels, and limited nanoparticle shelf‐life due to oxidation and aggregation. The authors emphasized future directions such as improving nanoparticle stability, integrating automated chemometric workflows, and developing portable SERS–coffee‐ring platforms for rapid, on‐site detection of nitrogen‐based adulterants in dairy supply chains (Hussain et al. 2019). Overall, Raman and SERS‐based approaches have demonstrated strong potential for detecting urea adulteration in milk, offering chemically specific, rapid, and minimally invasive analysis. Continued advances in signal enhancement, substrate robustness, and data‐driven analytics will be essential to improve sensitivity, reproducibility, and field deployability, thereby strengthening Raman spectroscopy's role in comprehensive dairy adulteration surveillance.

#### 4.2.3 Ammonium Sulfate (AmS)

AmS is occasionally added to milk as an economically motivated adulterant to falsely elevate apparent protein content in nitrogen‐based analytical assays. Such adulteration is particularly concerning because excessive ammonium intake can disrupt acid–base homeostasis, induce renal stress, and increase vulnerability in infants and young children. Consequently, developing sensitive and reliable analytical strategies for AmS detection is essential for food safety assurance and regulatory compliance (Mamgain et al. 2024). Raman spectroscopy, when coupled with SERS, has shown strong potential for detecting inorganic nitrogen adulterants such as AmS in complex dairy matrices. Hussain et al. (2019) reported the simultaneous detection of urea and AmS in milk using SERS enhanced by the coffee‐ring effect, employing both AuNPs and Au@AgNPs. Nanoparticle synthesis was confirmed by UV–Vis spectroscopy and transmission electron microscopy, ensuring stable plasmonic properties for SERS enhancement.

The coffee‐ring deposition process concentrated analytes at the droplet perimeter, effectively amplifying Raman signals and improving detection sensitivity. Distinct Raman bands were observed at approximately 610 and 980 cm^−1^ for AmS, corresponding to sulfate vibrational modes, alongside urea markers at 1001 and 1460 cm^−1^ and intrinsic milk bands. Quantitative calibration exhibited strong linearity (*R* ^2^ ≈ 0.98–0.99), with detection limits reaching approximately 5 mg dL^−1^, outperforming many conventional analytical techniques that rely on extensive sample pretreatment or chemical derivatisation. Despite these advantages, several challenges remain. Matrix‐induced signal suppression, nanoparticle instability over time, and reduced sensitivity near regulatory threshold concentrations continue to limit broader industrial adoption. Future research should focus on improving nanoparticle robustness and shelf‐life, integrating chemometric or deep‐learning‐based spectral analysis for ultra‐trace detection, and developing compact, portable SERS platforms capable of real‐time, on‐site monitoring. Such advancements would significantly enhance the applicability of Raman‐based methods for routine surveillance of inorganic nitrogen adulterants in milk and dairy supply chains (Hussain et al. 2019).

#### 4.2.4 Sodium Thiocyanate (STC) and Benzoic Acid (BA)

STC and BA are sometimes illicitly added to milk as chemical preservatives to suppress microbial growth and artificially extend shelf life, particularly under conditions of poor refrigeration or prolonged storage (Hussain et al. 2020; Yong et al. 2017). While effective as antimicrobial agents, excessive intake of these compounds poses significant health risks, including thyroid dysfunction, gastrointestinal irritation, allergic reactions, and potential toxicity affecting the liver, kidneys, and nervous system. Consequently, their detection is critical for ensuring dairy safety and regulatory compliance.

In Raman analysis, the diagnostic bands correspond to the SCN ion, which is the active species derived from STC upon dissolution. This distinction is important because the Raman signature originates from SCN ion rather than the sodium cation. Yong et al. (2017) demonstrated that Raman spectroscopy enables sensitive, label‐free quantification of SCN ion through its characteristic C≡N stretching vibration observed in the 2060–2120 cm^−1^ region. Additional spectral features from lactose (875–946 cm^−1^) and protein amide I–III bands (1230–1700 cm^−1^) provide contextual information, allowing discrimination between endogenous SCN naturally present in milk and exogenously added STC. As a nondestructive and rapid technique, Raman spectroscopy is well suited for high‐throughput dairy quality monitoring. However, practical challenges remain, including fluorescence interference, spectral overlap from the complex milk matrix, and reduced sensitivity near regulatory threshold concentrations. Moreover, natural variability associated with geographic origin, animal diet, and lactation stage necessitates robust calibration models and advanced chemometric analysis. Future efforts should focus on integrating SERS substrates, standardized spectral preprocessing, and portable microfluidic‐Raman platforms to improve sensitivity and enable real‐time, on‐site screening (Yong et al. 2017).

**FIGURE 5:**
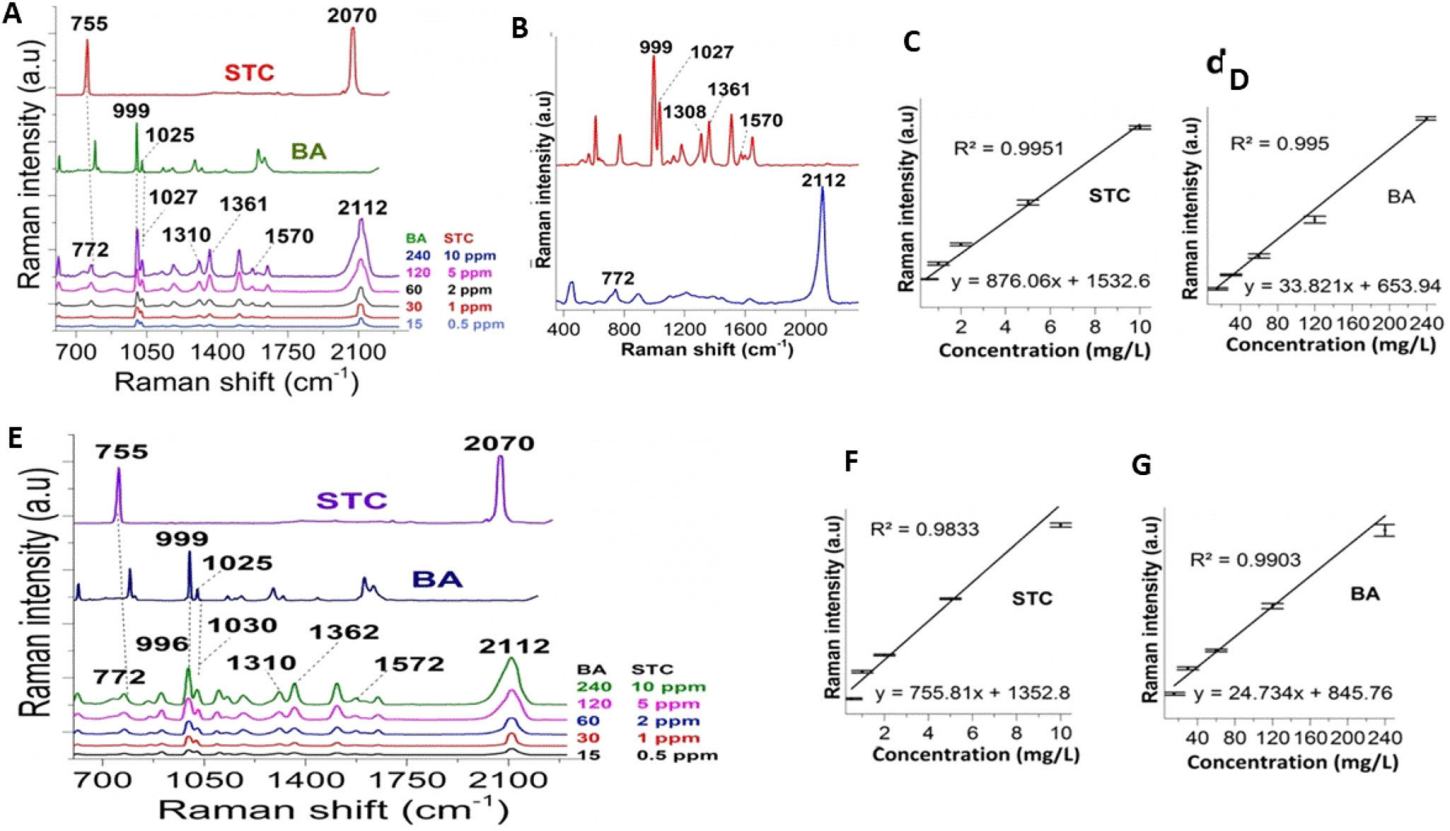
**Raman and SERS‐based detection of STC and BA in standard solutions and liquid milk**: (A) Raman spectra of solid STC and BA, along with combined spectra in standard solutions at varying concentrations, highlighting diagnostic peaks near 2070 cm^−1^ (C≡N stretch for SCN) and 755 cm^−1^ (C─S stretch) for STC, and aromatic ring bands for BA. (B) Key Raman bands selected for qualitative and quantitative analysis. (C and D) Calibration curves for STC and BA in standard solutions using peaks at 2112 cm^−1^ (STC) and 999 cm^−1^ (BA). (E) Raman spectra of STC and BA detected in spiked milk samples, showing slight peak shifts (e.g., STC from 2112 to ∼2070 cm^−1^) due to matrix effects and analyte–nanoparticle interactions. (F and G) Corresponding calibration curves for STC and BA in milk, demonstrating good linearity and sensitivity despite matrix complexity. BA, benzoic acid; STC, sodium thiocyanate. *Source*: Figure adapted from Hussain et al. 2020 with permission from Elsevier.

Building on these developments, Hussain et al. (2020) reported a highly sensitive SERS‐based approach for the simultaneous detection of STC and BA in liquid milk using cysteamine‐functionalized Au@Ag–CysNPs. The engineered nanoparticles provided abundant electromagnetic hot spots and enhanced analyte adsorption, resulting in strong Raman signal amplification. Characteristic STC peaks were observed at 2112 cm^−1^ (antisymmetric S─C≡N stretch) and 772 cm^−1^ (C─S symmetric stretch). In contrast, BA exhibited prominent aromatic ring deformation bands at 996 and 1030 cm^−1^, along with additional features at 1310, 1362, and 1572 cm^−1^. Quantitative analysis demonstrated excellent linearity (coefficient of determination (*R* ^2^) = 0.9833–0.9951) with low limits of detection of 0.03 mg L^−1^ for STC and 9.8 mg L^−1^ for BA in milk. Figure 5 illustrates Raman and SERS‐based detection of STC and BA, highlighting diagnostic spectral markers and calibration performance. Panels A, B, and E show Raman spectra for solid compounds, standard solutions, and spiked milk samples, where the C≡N stretch near 2070 cm^−1^ and C─S band at ∼755 cm^−1^ confirm STC, whereas BA is identified by aromatic ring deformation bands around 996–1030 cm^−1^. Panels C, D, F, and G present calibration curves for STC and BA in both standard solutions and milk, constructed from peaks at 2112 and 999 cm^−1^, respectively. Slight peak shifts in milk samples reflect matrix effects and analyte–nanoparticle interactions, yet quantitative models maintain strong linearity and sensitivity, demonstrating the robustness of SERS for simultaneous detection of STC and BA in complex dairy matrices (Hussain et al. 2020).

#### 4.2.5 Dicyandiamide (DCD)

DCD is a nitrogen‐rich compound that may be illicitly added to milk to artificially inflate apparent protein content or to conceal dilution, thereby exploiting nitrogen‐based quality assessment methods. The presence of DCD in dairy products is of significant concern due to its potential nephrotoxic and metabolic effects, as well as its broader implications for food safety and consumer trust, particularly in infant nutrition (Natarajan et al. 2023). Lin et al. (2015) reported a detailed Raman and SERS investigation of DCD, identifying strong and reproducible vibrational signatures at 147, 495, 661, 935, and 2161 cm^−1^. These assignments were rigorously validated through density functional theory calculations at the B3LYP/6‐31G(d) level, demonstrating excellent agreement between theoretical and experimental spectra and confirming the molecular origin of the diagnostic bands. Among these, the peaks at approximately 663 cm^−1^ (ring deformation), 926–935 cm^−1^ (C─N stretching), and 2161 cm^−1^ (C≡N stretching) were particularly informative for quantitative analysis. SERS enhancement was found to be strongly dependent on solution chemistry and nanoparticle aggregation behavior. Optimal signal amplification was achieved under neutral conditions in the presence of Cl^−^ ions, and under alkaline conditions using Na_2_SO_4_ and NaOH as aggregating agents. Using an internal standard band at 866 cm^−1^ to correct for signal fluctuations, DCD quantification in milk exhibited excellent linearity (*R* ^2^ ≈ 0.997) with limits of detection as low as 0.1 mg mL^−1^, enabling rapid screening without extensive sample pretreatment. These results highlight the suitability of SERS for routine monitoring of nitrogen‐based adulterants in dairy products (Lin et al. 2015).

Nevertheless, several analytical challenges remain. Citrate ions commonly present in milk can interfere with nanoparticle aggregation, whereas spectral overlap in the 922–935 cm^−1^ region complicates accurate peak assignment. In addition, pH‐dependent band shifts and sensitivity to salt concentration can affect reproducibility, as excessive Cl^−^ may saturate nanoparticle surfaces and suppress DCD adsorption. Future research should therefore focus on the development of engineered SERS substrates with enhanced hot spot density and controlled surface chemistry, integration of microfluidic‐Raman platforms for automated and reproducible analysis, and the application of AI‐assisted spectral deconvolution and chemometric modeling to mitigate matrix interference and enable ultra‐trace detection in real‐world milk supply chains (Lin et al. 2015). Table 4 summarizes Raman and SERS strategies for detecting common chemical adulterants in milk, including urea, AmS, thiocyanates, BA, and DCD. Section (A) lists the diagnostic vibrational bands that serve as molecular fingerprints for each adulterant, highlighting functional group‐specific modes such as C≡N stretching for DCD and COO^−^ scissoring for BA. Section (B) outlines representative experimental implementations, spanning benchtop and portable Raman systems as well as advanced SERS platforms employing AgNPs and Au@Ag–CysNPs for enhanced sensitivity. Together, these data illustrate the efficacy of Raman‐based techniques in combining spectral specificity with adaptable instrumentation, enabling rapid and reliable screening of adulterants in complex dairy matrices.

**TABLE 4:** Raman‐ and SERS‐based detection of chemical adulterants in milk: excitation conditions, enhancement strategies, and analytical performance.

(A) Key diagnostic Raman and SERS bands of chemical adulterants (Raman: Raman spectroscopy; SERS: surface‐enhanced Raman spectroscopy)		
Adulterant	Raman/SERS band (cm^−1^)	Vibrational assignment
Urea	1170	NH_2_ rocking vibration
	1460–1461	Antisymmetric N─C─N stretching
	1530–1636	NH_2_ deformation and C═O stretching
	1008	Symmetric N─C─N stretching
Ammonium sulfate (AmS)	976, 1008	SO_4_^2−^ symmetric stretching (characteristic band)
Thiocyanates (SCN)	442–445	S─C≡N bending vibration
	747–756	C─S stretching vibration
	755/772	C─S symmetric stretching
	2070–2112	S─C≡N antisymmetric stretching
Benzoic acid (BA)	996–1027	Benzene ring deformation
	1310	C─H in‐plane bending
	1361–1362	COO^−^ in‐plane scissoring
	1570–1572	C─C stretching (benzene ring)
Dicyandiamide (DCD)	2161–2198	C≡N/C═N stretching
	926–935	N─C─N and C─N stretching
	667	NH_2_ deformation
	495/661	N─C rocking and N─C≡N bending
	676	In‐plane ring breathing II (triazine ring)

(B) Representative Raman and SERS implementations in milk matrices	
Urea	Near‐infrared Raman spectroscopy (*λ* = 785 nm, *P* ≈ 80 mW) (Khan et al., 2015); coffee‐ring‐assisted SERS (*λ* = 785 nm) (Hussain et al. 2019)
Ammonium sulfate (AmS)	Confocal Raman spectroscopy (Renishaw system 1000; *λ* = 785 nm, *P*_sample = 2.5 mW, 50× objective) (Nieuwoudt et al. 2016b); portable Raman with fiber‐optic probe (*λ* = 785 nm, *P* = 200 mW) (Nieuwoudt et al. 2017); SERS with AgNPs (Yang et al. 2014)
Thiocyanates (SCN)	Portable Raman spectroscopy (*λ* = 785 nm); SERS with AgNPs in milk (Q. Yang et al. 2014); confocal Raman (*λ* = 633 nm) and SERS using Au@Ag–CysNPs (Hussain et al. 2020)
Benzoic acid (BA)	Confocal Raman spectroscopy (Horiba France SAS, *λ* = 633 nm, *P* = 50 mW); SERS with Au@Ag–CysNPs (Hussain et al. 2020)
Dicyandiamide (DCD)	Confocal Raman spectroscopy (Renishaw system 1000; *λ* = 785 nm, *P*_sample = 2.5 mW) (Nieuwoudt et al. 2016a); portable Raman spectroscopy (BWS415‐785H, *λ* = 785 nm, *P* = 150 mW) with AgNP‐based SERS (Lin et al. 2015)

*Note*: λ denotes excitation wavelength; P, laser power; t_exp, exposure time.

Collectively, studies on chemical adulterants, such as melamine, urea, AmS, STC, BA, and DCD, demonstrate the versatility and molecular specificity of Raman and SERS‐based approaches for safeguarding milk quality. The presence of well‐defined vibrational fingerprints, coupled with advances in nanostructured substrates, chemometric modeling, and deep learning, has enabled sensitive detection across a wide concentration range, even within the chemically complex milk matrix. Although chemical adulterants primarily exploit weaknesses in compositional testing, nutritional and compositional adulteration represents a subtler yet equally significant threat to milk integrity. The following section addresses these practices and the role of Raman spectroscopy in their detection.

### 4.3 Nutritional and Compositional Adulterants

Detection of nutritional and compositional adulteration in milk by Raman spectroscopy poses a subtle but significant threat to milk quality, involving deliberate manipulation of naturally occurring components to increase economic gain while evading routine quality control. Unlike chemical adulterants, these practices exploit the inherent variability of milk, making detection challenging. Raman spectroscopy, sensitive to molecular structure and conformation, provides a powerful tool for identifying these modifications by monitoring characteristic vibrational signatures of carbohydrates, proteins, and lipids.

**TABLE 5:** Raman spectroscopic analysis of nutritional and compositional components in milk.

Raman/SERS band (cm^−1^)	Assignments (functional groups)	Instrument/Experimental context
Lactose		
869	O─C─O deformation mode	Raman spectrometer (I‐Raman Plus, B∖&W Tek, Delaware, USA; *λ* = 785 nm, *P* = 100 mW) (Li et al. 2015)
940	C─C stretching	
1015	C─C stretching	
1085	C─O─C stretching	
1121	C─C stretching/aliphatic C─C contribution	
Whey proteins		
3350 and 3550	O─H stretching vibrations	FT‐Raman (Bruker RFS‐100; Nd:YAG laser, *λ* = 1064 nm, *P* = 300 mW, Δ*ν* ≈ 4 cm^−1^) (de Oliveira Mendes et al. 2020)
2894	H─C asymmetric stretching (lipids)	
2854	H─C symmetric stretching	
2888 and 2978	C─H stretching (lactose‐related)	
1656	C─N and C═C stretching (amide I, unsaturated fatty acids)	
1442	CH_2_ bending (scissoring, lipids)	
1303	CH_2_ bending (twisting)	
1120–850	C─O and C─H stretching; C─O─C deformation (lactose)	
Linoleic acid		
3005–3014	═C─H stretching	FT‐Raman, Nd:YAG laser (*λ* = 1064 nm) (El‐Abassy et al. 2011)
1652	C═C stretching (*cis*–*trans* conjugated)	
1440	CH_2_ bending	
1300–1310	CH_2_ twisting/wagging	

#### 4.3.1 Lactose

Detection and quantification of lactose in milk is critical for multiple reasons: ensuring accurate nutritional labeling, verifying product authenticity, supporting the production of lactose‐free or low‐lactose alternatives, and identifying adulteration practices such as dilution or substitution that may compromise milk quality and safety (Facioni et al. 2020). Precise lactose monitoring is particularly important for vulnerable populations, including infants, the elderly, and lactose‐intolerant individuals, for whom incorrect labeling could pose health risks. Raman spectroscopy offers a rapid, nondestructive, and label‐free approach for lactose analysis by exploiting the unique vibrational signatures of saccharides. Li et al. (2015) developed a method enabling rapid quantification of lactose using crystal violet as an internal standard, allowing precise normalization and improved analytical reliability. In this study, Raman spectra effectively differentiated lactose from other saccharides such as glucose and galactose within the 800–1300 cm^−1^ spectral range. Distinct lactose peaks were observed at 869, 940, 1015, 1085, and 1121 cm^−1^, with the 1085 cm^−1^ band chosen as the primary marker due to its strong concentration dependence and absence in lactose‐free milk. Incorporation of the internal standard crystal violet (1173 cm^−1^) further enhanced quantification robustness, achieving excellent linearity (*R* ^2^ = 0.9992) and a low detection limit of 0.019 mol L^−1^ (Li et al. 2015).

#### 4.3.2 Whey

Whey adulteration in milk, whether intentional or accidental, can alter nutritional composition, mislead quality assessment, and compromise product safety. Rapid detection and quantification are therefore essential for regulatory compliance and consumer protection. Raman spectroscopy has emerged as a powerful tool for identifying whey adulteration, leveraging its ability to capture molecular vibrational fingerprints of milk constituents. De Oliveira Mendes et al. (2020) demonstrated that Raman spectroscopy could effectively detect and quantify whey adulteration in raw cow's milk within a 0%–20% addition range. Key Raman bands corresponded to major milk components, including lactose, proteins, and fats: O─H stretching at 3350–3550 cm^−1^, lipid CH stretching at 2925 cm^−1^, amide I and unsaturated fatty acids at 1656 cm^−1^, CH_2_ bending at 1442 and 1303 cm^−1^, and lactose vibrations at 1120–850 cm^−1^. Despite the inherent spectral similarity between milk and whey, multivariate chemometric approaches such as PCA and PLSR enabled reliable discrimination and quantification. The partial least squares (PLS) model exhibited excellent predictive performance (*R* ^2^ > 0.98, RMSEP <1%, residual predictive deviation [RPD] >13) with no significant bias, indicating strong potential for accurate industrial monitoring (de Oliveira Mendes et al. 2020).

#### 4.3.3 Linoleic Acid (LA)

LA, a naturally occurring omega‐6 fatty acid in milk, is crucial for cellular function, lipid metabolism, and regulation of inflammation (Ju et al. 2025). However, the deliberate addition of synthetic or low‐quality vegetable oils can be used to artificially boost milk fat content and mimic natural lipid profiles, constituting a nutritional adulteration strategy (Meurens et al. 2005). While naturally occurring conjugated LA provides health benefits such as anti‐inflammatory effects and improved metabolism (Ju et al. 2025) excessive or synthetic LA adulteration can disrupt the omega‐6 to omega‐3 balance, increasing inflammation, cardiovascular risks, and compromising overall milk quality and digestibility (Anton 2013). El‐Abassy et al. (2011) applied Raman analysis to cow milk fat, identifying lipid‐associated bands that correlated proportionally with fat content (0.3%–4.0%), whereas protein and carbohydrate contributions remained comparatively weak. Key Raman peaks included 1650 cm^−1^ (C═C stretch), 1440 cm^−1^ (CH_2_ scissoring), 1265 cm^−1^ (*cis* C─H bending), 1300 cm^−1^ (CH_2_ twisting), and 1747 cm^−1^ (C═O ester stretch), with additional carotenoid features observed at 1008, 1150, and 1525 cm^−1^. PLSR applied across the 800–3050 cm^−1^ range demonstrated high predictive accuracy, particularly for samples analyzed in open aluminum dishes or as dried droplets on aluminum foil. Measurements using quartz cuvettes were less accurate due to scattering and surface turbidity, and low‐fat samples exhibited higher spectral noise. Despite these challenges, Raman spectroscopy provides a preparation‐free, nondestructive method for detecting both natural and adulterated LA in milk. Building on these considerations, the next section examines Raman‐based strategies for detecting biological adulterants and contaminants, specifically antibiotic residues and bacterial contamination which are critical for safeguarding dairy safety and protecting public health (El‐Abassy et al. 2011). Table 5 summarizes Raman‐based investigations of key nutritional and compositional components in milk, including lactose, whey proteins, and LA. The listed vibrational bands represent characteristic molecular fingerprints: Lactose exhibits strong C─O─C and C─C stretching modes, whey proteins display amide I and O─H stretching features, whereas LA is distinguished by unsaturated C═C and ═C─H stretching vibrations. Experimental configurations range from benchtop Raman systems to FT‐Raman platforms employing Nd:YAG excitation, demonstrating the versatility of Raman spectroscopy for probing both carbohydrate and lipid fractions in complex dairy matrices. These spectral markers reinforce quantitative and qualitative assessments of milk composition, supporting applications in nutritional profiling and authenticity verification.

### 4.4 Biological Contaminants

#### 4.4.1 Antibiotic Residues

Ampicillin residues in milk may arise from unregulated veterinary use or deliberate addition to inhibit bacterial growth and extend shelf life. Consumption of ampicillin‐contaminated milk poses significant health risks, including the promotion of antibiotic resistance, allergic reactions, and disruption of the gut microbiome, particularly in infants (Alenezi et al. 2024; Vardanyan and Hruby 2006). Andreou et al. (2015) developed a microfluidic SERS platform for rapid detection of trace ampicillin in milk. Characteristic Raman peaks corresponding to phenyl‐ring vibrations at 1007 and 1035 cm^−1^ and secondary amide vibrations at 1115 cm^−1^ enabled detection down to 10 ppb, whereas a SERS peak near 230 cm^−1^ indicated AgNP aggregation. The method offered fast, culture‐free screening with strong potential for portable, automated food‐safety monitoring (Andreou et al. 2015). Complementary work by Acar‐Soykut et al. (2018) demonstrated differentiation between ampicillin‐ and phage‐contaminated milk using Raman spectroscopy combined with PCA. Distinctive bands at 248, 350, 492, 1064, 1340, and 1367 cm^−1^, alongside lactose peaks at 875, 946, 1010, and 1125 cm^−1^, allowed detection at concentrations as low as 0.5 µg mL^−1^. Despite its sensitivity, the approach is challenged by spectral overlap in complex milk matrices and weak signals at very low phage titers (Acar‐Soykut et al. 2018).

#### 4.4.2 Bacterial Contamination

Milk can harbor pathogenic and spoilage bacteria, including *Brucella* spp., *Ochrobactrum*, *Lactococcus cremoris*, *Staphylococcus aureus*, and *Listeria monocytogenes*, each posing unique risks to food safety and human health (Bellio et al. 2016; Vasavada 1988; Zeb et al. 2025). Raman spectroscopy has been successfully applied to detect and differentiate bacterial species in milk. Meisel et al. (2012) demonstrated that *Brucella* spp. exhibited stronger DNA bands at 729, 781, 1487, and 1578 cm^−1^, cytochrome bands at 748, 1130, and 1578 cm^−1^, and lipid peaks at 2850 cm^−1^, distinguishing it from *Escherichia* spp., which showed prominent protein signals (1208–1675 cm^−1^) and RNA/DNA peaks (815, 1327 cm^−1^). *Ochrobactrum* spectra featured enhanced phenylalanine (1003 cm^−1^) and CH_2_ deformation (1448 cm^−1^) bands, whereas Listeria species exhibited consistent peaks at 724, 780, 1003, 1335, 1453, and 1667 cm^−1^. FT‐IR and Raman analyses revealed complementary molecular fingerprints of bacterial growth, enabling discrimination of species and mixed cultures. Chemometric analysis is often necessary for accurate classification due to spectral similarity among related species. These findings underscore Raman spectroscopy's potential for rapid, label‐free monitoring of microbial contamination in dairy products (Meisel et al. 2012). Table 6 consolidates Raman and SERS‐based strategies for detecting biological contaminants in milk, including antibiotic residues (e.g., ampicillin) and major bacterial pathogens such as *L. monocytogenes*, *Brucella* spp., *Escherichia* spp., *Pseudomonas* spp., *Yersinia* spp., and *S. aureus*. The listed vibrational bands correspond to key biomolecular signatures: nucleic acid markers (adenine, cytosine, and guanine), protein‐related amide bands, lipid‐associated CH_2_/CH_3_ vibrations, and phenylalanine ring modes. Experimental configurations span confocal micro‐Raman systems and SERS platforms employing AgNPs for enhanced sensitivity. These spectral fingerprints enable rapid, label‐free identification of microbial contaminants and antibiotic residues, supporting advanced quality assurance and food safety monitoring in dairy supply chains.

**TABLE 6:** Raman‐ andSERS‐based detection of biological contaminants in milk: diagnostic bands, biomolecular assignments, and instrumental configurations.

(A) Raman/SERS bands and assignments for biological contaminants in milk		
Antibiotic residues (Ampicillin)		
Raman/SERS band (cm^−1^)	Assignment	Biomolecular context
1007	Phenyl ring vibration	Core aromatic structure of β‐lactam antibiotic
1035	Amide II	Protein‐like vibration; drug backbone
1115	PDMS‐related band	Substrate signal (Si─O─Si stretch)
1415	Weak deformation mode	CH_2_/CH_3_ bending (matrix or drug)

Gram‐positive bacteria		
Raman/SERS band (cm^−1^)	Assignment	Biomolecular context
1003	Phenylalanine	Protein marker; robust bacterial fingerprint
780–781	Cytosine/uracil	DNA/RNA bases
1230	Amide III	Protein secondary structure
1335	CH_3_/CH_2_ modes	Lipid/protein side chains
1630–1656	Amide I	Protein backbone; unsaturated fatty acids
2850–2940	CH_2_/CH stretching	Lipids and fatty acids

Gram‐negative bacteria		
Raman/SERS band (cm^−1^)	Assignment	Biomolecular context
729	Adenine	DNA base
781	Cytosine/uracil	DNA/RNA
1094	DNA backbone	PO_2_^−^ phosphate vibrations
1487, 1578	Guanine	DNA marker
748	Cytochrome	Heme‐associated vibration
1130	Proteins/fatty acids	Mixed biomolecular contribution
1440	CH_2_ deformation	Lipid/protein
2940	C─H stretching	Lipids and proteins

(B) Instrumental configurations for Raman and SERS detection of biological contaminants in milk		
Code	Contaminant(s)	Instrument/Experiment
I1	Ampicillin	Confocal micro‐Raman (LabRam Aramis, Horiba); *λ* = 633 nm, *P* = 3.8 mW (Acar‐Soykut et al. 2018)
I2	Ampicillin	SERS with AgNPs in milk, microfluidic device (Andreou et al. 2015)
I3	*Listeria monocytogenes*	Confocal micro‐Raman with Leica microscope; *λ* = 785 nm, *P* = 300 mW (Wang et al. 2015)
I4	*Brucella* spp.	
I5	*Escherichia* spp.	
I6	*Pseudomonas*, *Yersinia* spp.	
I7	*Staphylococcus aureus*, *Lactococcus lactis* ssp. *cremoris*	Raman with diode laser; *λ* = 785 nm, *P* = 2 mW; ultraheated milk (Nicolaou et al. 2011)

*Note*: λ denotes excitation wavelength; P, laser power; PDMS, polydimethylsiloxane. SERS‐on‐chip with AgNPs (I2) enables rapid detection of ampicillin at trace levels in milk, achieving results in under 10 min with less than 20 µL of sample (Andreou et al. 2015). Chemometric micro‐Raman analysis at 785 nm (I3) discriminates Listeria species in milk with over 93%–96% accuracy, leveraging key spectral markers such as nucleic acids, phenylalanine, amide bands, and lipid‐associated vibrations (Wang et al. 2015). Extensive micro‐Raman studies at 532–785 nm (I4–I7) have been applied for single‐cell identification of pathogens including *Brucella*, *Escherichia*, *Pseudomonas*, *Yersinia*, and *Staphylococcus*, consistently highlighting diagnostic bands for DNA bases (adenine at 729 cm^−1^; cytosine/uracil at 781 cm^−1^; guanine at 1487 and 1578 cm^−1^), phenylalanine (1003 cm^−1^), amide regions, and lipid CH stretches (Meisel et al. 2012). Additionally, PDMS substrates used in microfluidic SERS devices exhibit characteristic Raman bands, including Si─O─Si stretching (∼1020–1090 cm^−1^) and CH_3_ deformation (∼1260 and ∼1410 cm^−1^). Importantly, the band near 1115 cm^−1^ may originate from PDMS rather than the analyte (e.g., ampicillin), making this distinction critical to avoid misinterpretation of spectra in milk analysis.

### 4.5 Emerging and Unconventional Adulterants

#### 4.5.1 Carbon Nanotubes (CNTs)

CNTs may enter milk through environmental contamination, packaging materials, or residues from processing equipment and have also been explored for antimicrobial applications in food preservation (Zeng et al. 2016). Ingestion of CNTs poses serious health risks, including oxidative stress, inflammation, potential carcinogenicity, and long‐term cellular and immune system damage. Nunes et al. (2024) demonstrated effective detection of multi‐walled CNTs in milk using Raman spectroscopy. Native milk spectra exhibited characteristic peaks at 2931 and 2856 cm^−1^ (CH_2_ stretching), 1655 cm^−1^ (amide I/protein–lipid), 1450 cm^−1^ (CH_2_ deformation), 1261 cm^−1^, 1122–1084 cm^−1^ (C─O/C─C), and 1003 cm^−1^ (phenylalanine). In contrast, multi‐walled CNTs produced distinct D (∼1286 cm^−1^) and G (∼1605 cm^−1^) bands, with intensity decreasing upon dilution. Using partial least squares discriminant analysis (PLS‐DA), the model achieved 100% sensitivity and 93%–100% specificity, with reliable detection down to 0.1 µg mL^−1^ (Nunes et al. 2024).

### 4.6 Raman‐Based Authentication of Liquid Milk—Summary and Outlook

The comprehensive body of work reviewed in this section highlights Raman spectroscopy as a robust, versatile, and increasingly mature analytical platform for detecting adulteration and contamination in liquid milk. Across cow and buffalo milk systems, Raman‐based approaches have demonstrated strong capability in identifying a wide spectrum of adulterants, including chemical additives (e.g., urea, AmS, STC, BA, and DCD), nutritional and compositional modifiers (lactose dilution, whey addition, and fatty‐acid manipulation), biological contaminants (antibiotic residues and pathogenic bacteria), and emerging unconventional threats such as CNTs. The molecular specificity of Raman vibrational fingerprints enables simultaneous interrogation of lipids, proteins, carbohydrates, and foreign substances within the complex milk matrix, offering a distinct advantage over conventional targeted assays. A key strength of Raman spectroscopy lies in its nondestructive, label‐free nature, minimal sample preparation, and compatibility with rapid, high‐throughput screening. Integration of chemometric tools, including PCA, PLS, PLS‐DA, and more recently ML and deep‐learning frameworks, has been critical in overcoming challenges related to spectral overlap, matrix interference, and natural compositional variability. These multivariate approaches enable both qualitative discrimination and quantitative estimation of adulterants at concentrations relevant to regulatory limits, even in highly heterogeneous liquid milk samples. Advances in SERS, microfluidic SERS platforms, and portable Raman instrumentation further expand applicability for on‐site monitoring and real‐time quality control in dairy processing environments. Based on these developments in liquid milk, the following section 5 explores Raman spectroscopic detection of adulteration in buffalo milk, highlighting species‐specific challenges, spectral markers, and advanced chemometric strategies for accurate and sensitive monitoring.

## 5 Raman Spectroscopic Detection of Adulteration in Buffalo Milk

Buffalo milk occupies a prominent position in the global dairy sector, particularly across South Asia, the Middle East, and parts of Europe, owing to its naturally higher fat, protein, and mineral content compared with cow milk (Chauhan and Selokar 2022). These compositional attributes contribute to superior nutritional value, richer sensory characteristics, and enhanced processing performance; however, they also substantially elevate the economic value of buffalo milk (Liao et al. 2025). As a consequence, buffalo milk is especially vulnerable to economically motivated adulteration. Common fraudulent practices include dilution with water, substitution with lower cost milks such as cow or soybean milk, and the addition of chemical substances, including urea, ammonium chloride, sodium bicarbonate, sodium citrate, and sucrose, to artificially restore perceived physicochemical quality (Trimboli et al. 2019). Such interventions compromise nutritional integrity, distort labeling accuracy, and pose significant health risks, particularly for infants, elderly consumers, and immunocompromised populations. The development of rapid, sensitive, and nondestructive analytical approaches for detecting adulteration in buffalo milk is therefore critical for consumer protection and regulatory enforcement (Liao et al. 2025; Trimboli et al. 2019).

Owing to the high fat content of buffalo milk, spectra are dominated by intense lipid‐associated signals, notably CH_2_/CH_3_ stretching vibrations in the 2850–2950 cm^−1^ region. Protein contributions are reflected in amide I near ∼1656 cm^−1^ and amide III between 1240–1300 cm^−1^, whereas carbohydrates such as lactose give rise to characteristic features around ∼854 and ∼1004 cm^−1^. Variations in relative intensity, band shape, and distribution across these regions provide sensitive indicators of dilution, compositional imbalance, and the presence of foreign additives, enabling both qualitative discrimination and quantitative estimation of adulterants within complex buffalo milk matrices. Li et al. (2026) systematically investigated buffalo milk adulteration using Raman spectroscopy integrated with advanced chemometric and deep‐learning methodologies. To mitigate scattering and baseline variability arising from matrix heterogeneity, multiplicative scatter correction (MSC) was applied during preprocessing. Multivariate classification with PLS‐DA enabled robust differentiation between pure buffalo milk and samples adulterated with cow milk, soybean milk, water, and a range of chemical additives. PCA revealed clear clustering, indicating that even subtle compositional changes introduced by adulteration yield reproducible spectral fingerprints. Discrimination was largely driven by alterations in lipid‐to‐protein ratios, attenuation of lactose‐associated bands following dilution, and the emergence or amplification of features associated with nitrogen‐rich adulterants (Li, Li, et al. 2026).

Beyond qualitative classification, Raman spectroscopy demonstrated strong capability for quantitative analysis of adulterants in buffalo milk. MSC preprocessed PLSR and CNNs achieved high predictive accuracy, with coefficients of determination (*R* ^2^) up to 0.97 and LOD as low as 17.4 mg kg^−1^ for selected adulterants. The superior performance of deep‐learning approaches highlights their effectiveness in capturing nonlinear spectral variations and complex matrix effects that are difficult to model using conventional linear techniques. Collectively, these results support Raman–chemometric and Raman–deep‐learning frameworks for high‐throughput screening and multi‐adulterant quantification under realistic industrial and regulatory conditions (Li, Li, et al. 2026).

In contrast to cow milk and milk powder, where Raman studies frequently focus on individual adulterants or specific contaminant classes, the current buffalo milk literature remains comparatively limited and is predominantly oriented towards multi‐adulterant detection. Most reports investigate several chemical and compositional adulterants simultaneously, employing chemometric and deep‐learning models to capture the spectral variability associated with buffalo milk's high fat and protein content. Consequently, adulterant‐specific Raman markers are less often reported in isolation; methodological performance, model robustness, and classification accuracy form the primary analytical emphasis. This reflects the present state of the field rather than any inherent limitation and underscores the need for buffalo milk‐specific studies targeting individual adulterants and biological contaminants under both controlled and real‐world conditions.

At the same time, several matrix‐specific challenges remain for Raman‐based detection in buffalo milk. The substantial lipid fraction often attenuates Raman signals from low‐level adulterants, reducing detectability and complicating quantitative analysis. Spectral overlap between endogenous constituents and chemically similar additives further hinders accurate peak assignment and limits model generalizability. Natural variability, arising from breed, feeding regime, lactation stage, and seasonal factors, may also affect calibration transfer and increase the risk of overfitting. Addressing these issues will require larger and more diverse calibration datasets, hybrid preprocessing pipelines (e.g., MSC combined with derivative or Savitzky–Golay smoothing), and complementary techniques such as SERS or spatially offset Raman spectroscopy to improve sensitivity and enable depth‐resolved profiling. Overall, Raman spectroscopy, when combined with chemometric and deep‐learning tools, represents a powerful and versatile analytical platform for detecting adulteration in buffalo milk. Its capacity for rapid, nondestructive, multicomponent analysis positions it as a promising solution for real‐time quality assurance, supply chain monitoring, and regulatory compliance. Continued methodological refinement and technological integration are expected to enhance applicability further, supporting safer dairy practices and reinforcing consumer confidence in buffalo milk products. Building on these advances in liquid milk systems with high fat and protein content, the next section addresses Raman spectroscopic analysis of milk powder, where differences in physical state, processing history, and compositional concentration introduce both new analytical opportunities and distinct challenges for adulteration detection.

## 6 Raman Analysis of Milk Powder

Milk powder is a globally traded dairy product with extended shelf life and wide application in infant nutrition, clinical formulations, and food processing. Its high economic value and complex composition make it particularly vulnerable to adulteration and mislabeling. This section reviews Raman‐based strategies for species authentication, adulterant detection, and structural monitoring in milk powders.

### 6.1 Identification of Animal Origin in Milk Powder

**FIGURE 6:**
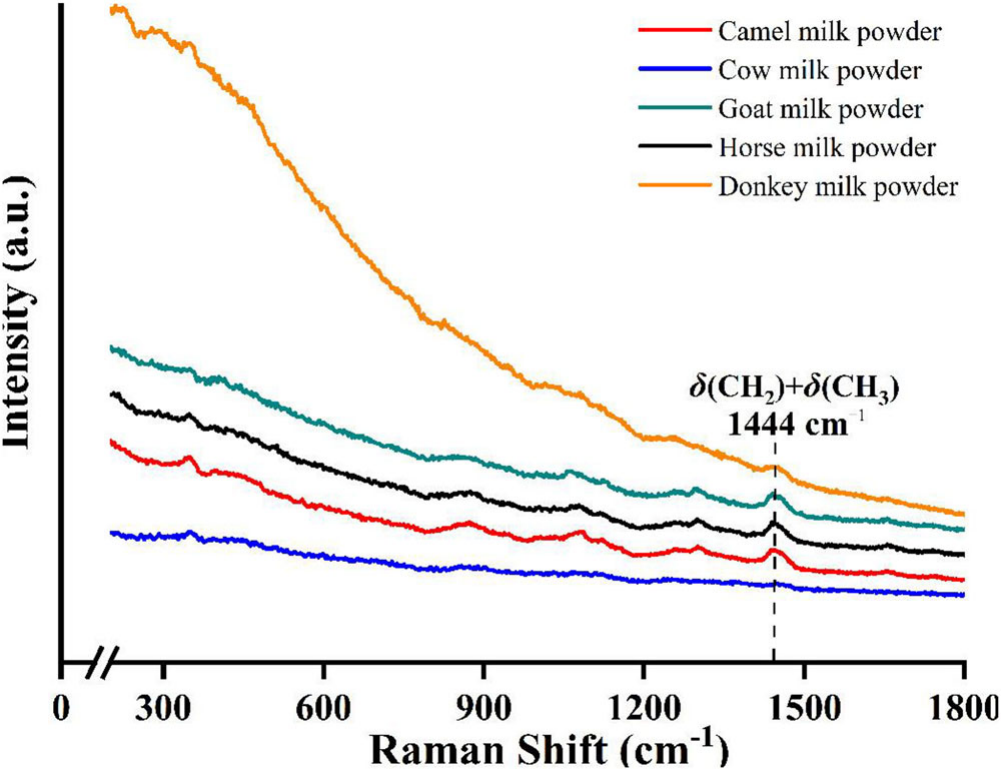
**Species‐specific Raman spectra of milk powders**. Raman spectra of milk powders derived from camel, cow, goat, horse, and donkey, recorded in the 200–1800 cm^−1^ fingerprint region. Prominent vibrational features correspond to major milk constituents, including proteins (amides I and III), lactose, and lipids. The intense band near 1444 cm^−1^, assigned to CH_2_ and CH_3_ bending vibrations in lipids, highlights interspecies differences in fat composition. Variations in band positions and relative intensities reflect compositional diversity among species, forming the basis for chemometric classification and authentication. *Source*: Reproduced from *Journal of Dairy Science* Zhang et al. 2025 with permission from American Dairy Science Association.

Accurate identification of the animal source of milk powder is critical for ensuring product authenticity, correct nutritional and allergenic labeling, religious and ethical compliance, and consumer trust. Raman spectroscopy has demonstrated strong potential for differentiating milk powders derived from different animal species based on variations in fat, protein, and carbohydrate composition. Zhang et al. (2025) reported a rapid Raman‐based approach for identifying milk powders from cow, goat, camel, horse, and donkey. The most informative spectral features were observed in the 400–1800 cm^−1^ region, corresponding to proteins (amide I and III), lactose, and lipids. A prominent band at approximately 1444 cm^−1^, arising from ─CH_2_ and ─CH_3_ bending vibrations in lipids, reflected interspecies differences in fat composition and content. However, substantial spectral overlap among species limited direct visual discrimination using raw spectra alone. Figure 6 illustrates species‐specific Raman profiles of milk powders in the 400–1800 cm^−1^ fingerprint region, highlighting compositional differences such as the prominent lipid‐associated band near 1444 cm^−1^, which underpins chemometric discrimination among cow, goat, camel, horse, and donkey samples (Zhang, Yang, et al. 2025).

To overcome this limitation, chemometric and ML models were employed. Multiclass classification algorithms, particularly multi class classifier and random forest, achieved high sensitivity, specificity, accuracy, and area under the curve values, enabling reliable qualitative identification and detection of mixed or adulterated milk powders. Quantitative estimation of adulteration ratios using PLSR, SVR, and principal component regression models was feasible, although predictive performance varied across milk types, with *R* ^2^ values frequently below 0.9, indicating challenges in model robustness and generalizability. These findings highlight the need for advanced preprocessing, optimized feature selection, and incorporation of species‐specific biomarkers, such as β‐lactoglobulin signatures in camel milk to improve predictive accuracy. Integration of deep‐learning frameworks is expected to further enhance real‐time authentication of animal‐derived milk powders in industrial and regulatory contexts (Zhang, Yang, et al. 2025). Beyond species authentication, Raman spectroscopy has also been widely applied to detect adulteration in milk powders, addressing chemical, compositional, and multi‐adulterant scenarios that pose significant safety and economic concerns.

### 6.2 Detection of Adulteration in Milk Powder

Milk powder adulteration is a major food‐safety and economic concern, often involving nitrogen‐rich chemicals, low‐cost carbohydrate fillers, or processing by‐products to mimic protein content and extend shelf life. These practices compromise nutritional integrity and regulatory compliance, posing severe health risks, particularly in infant formula markets where global trade amplifies vulnerability (Choudhary and Sharma 2024). Raman spectroscopy, especially when combined with SERS, advanced chemometrics, and hyperspectral Raman imaging (HSI), offers a rapid, nondestructive solution for detecting and quantifying such interventions across realistic concentration ranges in complex powdered matrices. Reliable performance depends on disciplined preprocessing, robust chemometric modeling, and validation under matrix‐matched conditions to ensure reproducibility and regulatory compliance.

#### 6.2.1 Detection of Nitrogen‐Rich Adulterants

Melamine detection has been achieved using cyclodextrin‐functionalized Ag nanoparticles, with diagnostic Raman bands near 381, 584, 676, and 984 cm^−1^; a ring‐breathing shift from ∼676 to ∼682 cm^−1^ indicates substrate interaction (Ma et al. 2013). DCD and AmS have been resolved in multicomponent powders using HSI with self‐modeling mixture analysis, exploiting C≡N bands (∼212 and ∼2200 cm^−1^) and sulfate markers (∼973 cm^−1^), alongside urea (∼1009 cm^−1^), down to ∼0.1%–5% (w/w). Spatial mapping clarifies heterogeneity even under fluorescence‐prone conditions (Qin et al. 2013). Push‐broom HSI with adaptive fluorescence correction extended melamine and urea detection in skimmed milk powder to ∼50 ppm, achieving strong pixel‐intensity correlations (*r* = 0.997) for high‐throughput screening (Qin et al. 2017). Complementary SERS workflows enable simultaneous detection of SCN and melamine using AgNPs with NaCl/NaOH, delivering excellent linearity (*R* ^2^ > 0.998) and high recoveries; SCN bands appear near ∼445 and ∼2060–2120 cm^−1^ (Yang et al. 2014).

#### 6.2.2 Carbohydrate Fillers and Whey

Economic adulteration with carbohydrate fillers and whey alters saccharide/protein signatures. Whey‐rich powders show pronounced lactose‐associated changes (e.g., 2978, 2888, 1087, and 850 cm^−1^), whereas starch exhibits a distinctive glycosidic ring band (∼477 cm^−1^). Perturbations near ∼1340 and ∼1080 cm^−1^ support discrimination at >5%, though lower levels require multivariate analysis and imaging‐based strategies (de Almeida et al. 2012). FT‐Raman distinguishes maltodextrin/modified lactose additions via lactose (2852, 1086, and 357 cm^−1^), glucose (2866, 1103, and 525 cm^−1^), and maltodextrin/starch (475–485 cm^−1^) markers, with PCA/PLS‐DA achieving 100% classification accuracy and detection limits near ∼10% (w/w) (Rodrigues Júnior et al. 2016).

#### 6.2.3 Structurally Similar Adulterants

Temperature‐dependent hyperspectral Raman differentiates structurally similar adulterants through phase‐transition and melting‐related band shifts corroborated by DSC. Discrimination among urea, biuret, cyanuric acid, and melamine at ∼1% (w/w) has been demonstrated, though requiring additional instrumentation and control (Schmidt et al. 2015).

#### 6.2.4 Process Monitoring and Lactose Hydrolysis

For lactose‐free formulations and storage stability, Raman enables process monitoring by tracking decreases in lactose‐specific modes (∼477, 377 cm^−1^) and increases in glucose/galactose bands (∼543, 410 cm^−1^), complemented by broader changes across 3000 cm^−1^ (C─H stretching), 1500–1250 cm^−1^ (CH_2_ deformation), and 1200–950 cm^−1^ (C─C/C─O stretching). Robustness benefits from humidity control and ageing studies to anticipate drift and ensure calibration transfer (Torres et al. 2017).

## 7 Fluorescence Interference and Spectral Challenges in Raman Analysis of Milk

Raman spectroscopy offers exceptional molecular specificity for milk authentication; however, fluorescence interference is a persistent challenge, particularly in complex liquid matrices rich in fats, proteins, pigments, and minor bioactives. Fluorescence can obscure weak bands, distort baselines, and reduce sensitivity, necessitating case‐specific mitigation via both experimental design and computational correction. Early mitigation strategies focused on NIR excitation, where reduced electronic absorption lowers fluorescence background. For example, 830 nm excitation enabled quantitative analysis of casein, lactose, and fatty acids in human breast milk within 600–1800 cm^−1^ (Motta et al. 2012). Although NIR significantly reduced autofluorescence, spectral overlap among milk components still limited resolution, requiring chemometric assistance. Similarly, SVM‐assisted classification achieved ∼86% accuracy in differentiating biochemical variations in breast milk (Ullah et al. 2018), yet fluorescence‐driven noise persisted in fatty acid‐rich regions, highlighting the limits of wavelength selection alone. Spatially resolved approaches such as confocal Raman microscopy and Raman imaging further mitigate fluorescence. Depth‐selective mapping can isolate lipid phase transitions and structural heterogeneity (1060–3010 cm^−1^), outperforming bulk measurements in fluorescence‐dense samples (de Wolf et al. 2021; Noda et al. 2007). These results emphasize that spatial discrimination can decouple Raman signals from luminescent backgrounds in heterogeneous matrices.

Fluorescence challenges are pronounced in contaminant/adulterant screening. Microplastic detection benefits from inherently low fluorescent polymer signatures within 841–1450 cm^−1^ (Ragusa et al. 2022), whereas antibiotic detection often requires SERS to overpower luminescent backgrounds. SERS limits down to 10^−9^ M for antibiotics (Mou et al. 2024) demonstrate that plasmonic enhancement can effectively suppress fluorescence via extreme signal amplification. Similarly, melamine in cow milk required dilution‐assisted SERS to reveal masked features (Yang et al. 2014), whereas engineered Au@SiO_2_ substrates reduced fluorescence and improved spectral stability (Li, Yang, et al. 2016), underscoring substrate design as a central mitigation strategy. Recent advances show that artificial intelligence (AI) can extract chemically meaningful information under high fluorescence conditions. Deep learning‐assisted Raman achieved 94.6% accuracy in detecting urea, melamine, and water adulteration (Liu et al. 2025). Quantitative goat milk adulteration studies using PCA/PLSR confirm robust performance despite fluorescence contributions from fat globules and complex matrices (Li et al. 2022; Zhu et al. 2024). Collectively, these studies establish fluorescence as a manageable parameter, not a fixed limitation. Through optimized excitation choice, advanced SERS substrates, confocal mapping, disciplined preprocessing, and AI‐based baseline reconstruction, Raman spectroscopy is increasingly capable of routine, high‐sensitivity detection of nutritional components, microplastics, antibiotics, and adulterants in real‐matrix milk systems.

## 8 Chemometric and Multivariate Data Analysis in Raman‐Based Milk Authentication

Raman spectra of milk contain rich molecular information, yet the inherent complexity of dairy matrices makes direct interpretation challenging (Joolaei Ahranjani et al. 2025). Overlapping bands from fats, proteins, and carbohydrates, compounded by baseline drift, fluorescence, and sample‐to‐sample variability, can obscure subtle adulteration signals. Consequently, chemometrics and multivariate analysis have become indispensable in Raman‐based milk authentication, enabling robust extraction of chemical, compositional, and classification information from complex spectral datasets (Ait El Alia et al. 2025; Windarsih et al. 2021).

### 8.1 Importance of Spectral Preprocessing

Reliable chemometric performance hinges on disciplined preprocessing. Raw milk spectra are frequently affected by baseline drift, scattering from fat globules, laser intensity fluctuations, and instrument noise. Common corrective steps include baseline correction, normalization, standard normal variate, and MSC to minimize nonchemical variation (Grassi et al. 2022). In many adulteration studies, MSC is particularly effective in stabilizing spectra impacted by turbidity and particle size variation, improving both classification and prediction. Derivative processing (first or second derivative) is often introduced when adulterant signals are weak or masked, enhancing resolution where protein and carbohydrate bands overlap (Grassi et al. 2022; Li, Pang, et al. 2026; Tian et al. 2022).

### 8.2 Exploratory and Unsupervised Analysis

Unsupervised tools, especially PCA, are widely used as the first analytical step. PCA reduces dimensionality while preserving dominant sources of variance, thereby revealing natural groupings, trends, and anomalies. In milk authentication, PCA consistently separates pure and adulterated samples based on differences in lipid‐, lactose‐, or protein‐associated bands (Li et al. 2022). Although PCA is not quantitative per se, loading plots provide chemical insight by highlighting spectral regions driving discrimination, making PCA valuable for understanding adulteration mechanisms and guiding marker selection (Ait El Alia et al. 2025; Grassi et al. 2022; Li et al. 2022).

### 8.3 Supervised Classification and Quantification

For definitive identification and quantification, supervised models are indispensable. PLS‐DA is widely used to classify samples by adulterant type, often achieving high sensitivity and specificity in multi‐adulterant systems (Tian et al. 2022). For concentration estimation, PLSR remains the most established method, supporting accurate prediction of water, whey, starch, urea, sugars, and chemical additives across broad ranges (Nieuwoudt et al. 2016b). Alternatives such as linear discriminant analysis, SVM, and *k*‐nearest neighbors are also effective; notably, SVM performs strongly where relationships are nonlinear or fluorescence interference persists (Ni et al. 2023; Ullah et al. 2018). Together, these supervised techniques move Raman beyond qualitative screening towards reliable, quantitative assessment.

### 8.4 Emergence of Machine Learning and Deep Learning Approaches

Recent work increasingly complements classical chemometrics with ML and deep learning. Neural architectures, such as CNNs and hybrid chemometric‐CNN frameworks, learn discriminative features directly from spectra, improving performance in heterogeneous milk matrices (Hu et al. 2022; Li, Li, et al. 2026). These approaches have enabled simultaneous detection of multiple adulterants at low levels and demonstrated greater robustness to fluorescence, baseline variation, and instrument drift attributes critical for real‐time and inline Raman applications outside tightly controlled laboratory environments.

### 8.5 Model Validation and Practical Robustness

Despite strong performance in development studies, models must be rigorously validated to ensure reliability. Cross validation and external validation using independent sets are essential, given natural variability introduced by animal breed, diet, season, and processing. Without careful validation, models risk overfitting and poor transferability across laboratories or instruments. Emerging strategies, multi‐batch calibration, transfer learning, and data augmentation are increasingly explored to enhance robustness and broaden applicability.

### 8.6 Relevance for Industry and Regulation

Integrating chemometrics with Raman spectroscopy enhances suitability for industrial and regulatory use. Automated data analysis reduces operator dependence and subjective interpretation, enabling rapid, reproducible decision‐making (di Donato et al. 2023; Grassi et al. 2022; Windarsih et al. 2021). Embedded within portable or inline Raman systems, validated models support real‐time screening of raw milk, processed products, and milk powders, reinforcing compliance with food safety regulations and fraud prevention initiatives. This capability is particularly valuable for large‐scale supply chains requiring high throughput and minimal sample preparation.

### 8.7 Emerging Chemometric and Explainable AI Strategies for Raman‐Based Milk Authentication

Future progress in Raman‐based milk authentication is expected to arise from the strategic integration of classical chemometric frameworks with advanced deep learning methodologies. Although deep learning offers superior predictive capability for complex and high‐dimensional spectral data, classical chemometrics remains indispensable for model transparency and chemical interpretability. In this context, explainable AI is poised to become a critical enabling technology, providing mechanistic insight by explicitly linking classification and prediction outcomes to chemically relevant Raman features and vibrational modes (Kalatzis et al. 2025). As curated Raman spectral libraries continue to grow in scale, diversity, and representativeness, and as models are trained on datasets spanning different milk types, processing conditions, and adulteration scenarios, chemometrics will increasingly function as the interpretative interface between raw spectral measurements and decision‐ready outputs. This evolution is particularly significant for regulatory and industrial adoption, where transparency, traceability, and auditability are essential. Collectively, these developments position chemometrics as a foundational component for scalable, robust, and regulator‐aligned milk authentication systems based on Raman spectroscopy (Kalatzis et al. 2025; Rossberg et al. 2025).

## 9 Future Perspectives and Translational Potential

The translational future of Raman‐based milk authentication points towards intelligent, adaptive, and field‐deployable systems that deliver laboratory‐grade specificity at the point of need while remaining robust to real‐matrix variability. Instrumentation will likely converge on multi‐excitation architectures capable of switching between 532, 633, 785, and 1064 nm to balance resonance enhancement against fluorescence suppression and penetration depth (de Oliveira Mendes et al. 2020; Motta et al. 2012; Ullah et al. 2018; Yang et al. 2014). In practical terms, 785 nm offers favorable detector efficiency with reduced autofluorescence for liquid milk, whereas 1064 nm (FT‐Raman) is advantageous for turbid, highly scattering matrices, including buffalo milk and milk powders, albeit at higher power and lower inherent sensitivity (Boyaci et al. 2015; de Almeida et al. 2012). A systematic wavelength analyte map linking excitation choice to pigments, antibiotics, SCN, nitrogen‐rich adulterants (e.g., urea, melamine, AmS), preservatives (e.g., benzoate), and lipid profiles would allow automatic wavelength selection to maximize signal‐to‐noise for each target within a single device (Wang, Li, et al. 2021). Complementary optical strategies such as spatially offset Raman spectroscopy for subsurface interrogation and hyperspectral Raman imaging for spatial heterogeneity will further support multi‐adulterant detection and distribution mapping in powders and complex liquid matrices (Dalal et al. 2025).

On the materials side, the field is moving beyond target‐specific substrates towards universal, multifunctional SERS architectures that integrate plasmonic metals (Au, Ag), dielectric shells (SiO_2_, Al_2_O_3_) for aggregation control and fouling resistance, polymer scaffolds for mechanical stability, and molecular recognition elements (aptamers and small molecule ligands) for selectivity (Guo et al. 2023; Krafft et al. 2020; Lin et al. 2015; Yang et al. 2021). The translational promise lies in embedding these substrates into paper strips, flexible swabs, and microfluidic chips, enabling genuine drop‐and‐measure workflows without specialist training (Lin et al. 2023; Tadi et al. 2024). For dairy‐grade deployment, substrate design must prioritize hot spot density, batch‐to‐batch reproducibility, shelf life, and matrix‐tolerant surface chemistry that resists competitive adsorption by proteins, lipids, and lactose (Tian et al. 2025). In parallel, microfluidic SERS platforms can precondition samples (filtration, phase separation, desalting) to reduce fluorescence and scattering, standardize residence times, and deliver reproducible enhancement in high‐fat systems such as buffalo milk (Krafft et al. 2020).

Future instruments should implement adaptive laser power control, dynamically modulating illumination to account for opacity, scattering efficiency, and thermal sensitivity, thereby amplifying weak adulterant bands while avoiding photothermal artifacts and peak shifts. This is particularly relevant for lipid‐rich matrices (where CH stretching modes near 2850–2950 cm^−1^ are prone to thermally induced line shape changes) and for powdered systems with variable multiple scattering (Aleksandar Nedeljković 2019; Silva et al. 2021). Adaptive power can be coupled to AI‐driven feedback from real‐time spectral quality metrics (baseline stability, outlier detection, and drift indices) to maintain analytical performance under field conditions (Coca‐Lopez et al. 2025). To support inline and handheld use, ruggedized optics, vibration‐resistant mounts, and clean‐in‐place compatible sample interfaces are necessary to meet hygiene standards and minimize downtime in processing environments. The data layer will increasingly anchor translational success. Automated chemometrics (baseline correction, SNV, MSC, and Savitzky–Golay smoothing) should be embedded and locked as standard operating procedure grade pipelines, ensuring consistent preprocessing across instruments and sites (Grassi et al. 2022; Li et al. 2022; Windarsih et al. 2021). Supervised models (PLS‐DA for classification; PLSR for quantification) should be complemented by ML/deep learning architectures (e.g., CNNs, attention networks) to capture nonlinearities and matrix effects that challenge classical linear methods (Li, Li, et al. 2026; Nisa et al. 2025). For industrial reliability, models must undergo rigorous cross‐validation, external validation, and matrix‐matched calibration, with performance reported using *R* ^2^, root mean square error of calibration /root mean square error of cross‐validation /RMSEP, bias, RPD, LOD/limit of quantification (LOQ), and area under the receiver operating characteristic curve for classifiers (Faust et al. 2022; Fearn 2002). To mitigate instrument‐to‐instrument variability and site‐specific drift, calibration transfer strategies, such as piecewise direct standardization, external parameter orthogonalization, and transfer learning, should be operationalized, supported by data augmentation that realistically simulates fluorescence, baseline curvature, and noise. As models become central to regulatory decisions, explainable AI will be critical for linking classifications and predictions back to chemically meaningful Raman features (e.g., triazine modes near ∼680–984 cm^−1^ for melamine, SCN, C≡N stretch near ∼2060–2120 cm^−1^, urea ∼1001–1461 cm^−1^), building trust among auditors and process engineers (Kalatzis et al. 2025; Rossberg et al. 2025).

Translational pathways must respect adulteration versus contamination as defined in this review: Adulteration is intentional addition/substitution for economic gain, whereas contamination is unintended introduction of biological, chemical, or physical hazards during production, processing, or storage. In practice, this distinction informs model objectives, decision thresholds, and escalation workflows. For regulatory alignment, LOD/LOQ targets should be benchmarked against Codex, EU, United States, and Asian limits (e.g., melamine thresholds in infant formula and other foods), and validation must include precision, accuracy, recovery, and robustness under realistic storage/transport conditions (Faust et al. 2022; Massarini et al. 2015). Buffalo milk warrants species‐specific calibration owing to its high fat and protein content; preprocessing hybrids (MSC with derivatives) and, where necessary, SERS or spatially offset Raman spectroscopy can bolster sensitivity for trace adulterants that are otherwise masked by endogenous bands (Li, Li, et al. 2026; Saleem et al. 2021; Trimboli et al. 2019; Ullah et al. 2017). Human breast milk applications will require ethical protocols, ultra ‐trace detection (SERS for antibiotics), and spectral libraries for polymer fingerprints to monitor microplastics. Milk powder workflows will benefit from 1064 nm FT‐Raman to suppress fluorescence, pressed pellet or thin film presentation to minimize multiple scattering, and HSI to map adulterant heterogeneity, all tied to chemometric pipelines that report concentration estimates and spatial distribution simultaneously (Boyaci et al. 2015; de Almeida et al. 2012; Mazurek et al. 2015).

Deployment at scale depends on device modularity and interoperability. A credible handheld platform would integrate multi‐wavelength excitation, interchangeable SERS cartridges, adaptive power, on‐device chemometrics/ML, and secure cloud‐connected spectral libraries with instrument‐agnostic data formats and audit trails (Byram et al. 2019; Mccain et al. 2008; Xiao et al. 2025). For factories, inline Raman scanners positioned at critical control points could support continuous surveillance, screening incoming tanker trucks, monitoring standardization/blending, and verifying powder lots before packaging (Tamer et al. 2024). Lifecycle management, including routine performance verification (wavelength calibration, intensity checks, and resolution metrics), drift monitoring, and scheduled recalibration with matrix‐matched standards, is essential to maintain analytical fidelity. Organizationally, successful translation requires cross‐disciplinary collaboration among spectroscopists, nanomaterials scientists, dairy technologists, and ML/AI experts, coupled with engagement from quality assurance teams, regulatory bodies, and standards organizations (e.g., AOAC/ISO) to codify method validation and calibration transfer guidelines for Raman in dairy authentication (Coca‐Lopez et al. 2025; Hu et al. 2022; Ullah et al. 2018).

Finally, cost–benefit clarity will accelerate adoption. Although capital expenditure for multi‐excitation Raman and high‐quality SERS substrates remains nontrivial, gains in throughput, reagent‐free operation, multi‐analyte coverage, and on‐site decision‐making can reduce laboratory burden and shorten response times in complex supply chains. As nano‐engineered substrates stabilize, spectral libraries expand, and embedded analytics mature, Raman spectroscopy is poised to shift from a predominantly laboratory method to a consolidated, field‐ready detection platform for milk and milk powder, strengthening consumer trust, supporting enforcement, and enhancing global dairy safety and authenticity. Against this backdrop of emerging technologies and translational strategies, the following conclusions distil the key insights and implications of this review.

## 10 Conclusions

Milk and milk powder remain central to global nutrition and trade, yet their vulnerability to adulteration and contamination poses persistent challenges for food safety, economic integrity, and consumer trust. Conventional analytical methods, though highly sensitive, are often fragmented, reagent‐intensive, and unsuitable for rapid, multi‐adulterant screening across complex supply chains. This review highlights Raman spectroscopy, particularly when enhanced through SERS and integrated with chemometrics and AI‐driven analytics, as a powerful alternative. Its ability to deliver molecular fingerprints, resolve vibrational features of proteins, fats, and carbohydrates, and detect adulterant‐specific bands at trace levels positions, thereby positioning Raman spectroscopy as a rapid, nondestructive, and high‐throughput solution. Advances in nano‐engineered substrates, fluorescence mitigation, and ML pipelines have significantly improved sensitivity and robustness, paving the way for real‐time, on‐site authentication. Looking ahead, the convergence of multi‐excitation instrumentation, universal SERS substrates, adaptive laser control, and cloud‐connected analytics will enable portable and inline Raman systems capable of screening multiple adulterants within seconds. Such platforms promise to strengthen regulatory compliance, industrial quality assurance, and consumer confidence, while reducing reliance on centralized laboratories. As these innovations mature, Raman spectroscopy is poised to evolve from a research tool into a consolidated detection framework, safeguarding milk quality across production, processing, transportation, and retail, supporting global food safety and authenticity.

## Author Contributions

**B. Sudarshan Acharya**: data curation, writing – original draft. **Sreerag Nair**: data curation, writing – original draft. **Abdul Ajees Abdul Salam**: conceptualization, writing – review and editing, supervision.

## Conflicts of Interest

The authors declare no conflicts of interest.
